# The impact of pictorial health warnings on purchases of sugary drinks for children: A randomized controlled trial

**DOI:** 10.1371/journal.pmed.1003885

**Published:** 2022-02-01

**Authors:** Marissa G. Hall, Anna H. Grummon, Isabella C. A. Higgins, Allison J. Lazard, Carmen E. Prestemon, Mirian I. Avendaño-Galdamez, Lindsey Smith Taillie

**Affiliations:** 1 Department of Health Behavior, Gillings School of Global Public Health, University of North Carolina at Chapel Hill, Chapel Hill, North Carolina, United States of America; 2 Lineberger Comprehensive Cancer Center, University of North Carolina at Chapel Hill, Chapel Hill, North Carolina, United States of America; 3 Carolina Population Center, University of North Carolina at Chapel Hill, Chapel Hill, North Carolina, United States of America; 4 Department of Nutrition, Harvard TH Chan School of Public Health, Boston, Massachusetts, United States of America; 5 Department of Population Medicine, Harvard Medical School and Harvard Pilgrim Health Care Institute, Boston, Massachusetts, United States of America; 6 Hussman School of Journalism and Media, University of North Carolina at Chapel Hill, Chapel Hill, North Carolina, United States of America; 7 Department of Nutrition, Gillings School of Global Public Health, University of North Carolina at Chapel Hill, Chapel Hill, North Carolina, United States of America; University of Cambridge, UNITED KINGDOM

## Abstract

**Background:**

Pictorial warnings on tobacco products are promising for motivating behavior change, but few studies have examined pictorial warnings for sugary drinks, especially in naturalistic environments. This study aimed to examine the impact of pictorial warnings on parents’ purchases of sugary drinks for their children in a naturalistic store laboratory.

**Methods and findings:**

Parents of children ages 2 to 12 (*n =* 325, 25% identifying as Black, 20% Hispanic) completed a shopping task in a naturalistic store laboratory in North Carolina. Participants were randomly assigned to a pictorial warnings arm (sugary drinks displayed pictorial health warnings about type 2 diabetes and heart damage) or a control arm (sugary drinks displayed a barcode label). Parents selected 1 beverage and 1 snack for their child, as well as 1 household good; one of these items was selected for them to purchase and take home. The primary outcome was whether parents purchased a sugary drink for their child. Secondary outcomes included reactions to the trial labels, attitudes toward sugary drinks, and intentions to serve their child sugary drinks. Pictorial warnings led to a 17-percentage point reduction in purchases of sugary drinks (95% CI for reduction: 7% to 27%), with 45% of parents in the control arm buying a sugary drink for their child compared to 28% in the pictorial warning arm (*p* = 0.002). The impact of pictorial warnings on purchases did not differ by any of the 13 participant characteristics examined (e.g., race/ethnicity, income, education, and age of child). Pictorial warnings also led to lower calories (kcal), purchased from sugary drinks (82 kcal in the control arm versus 52 kcal in the pictorial warnings arm, *p* = 0.003). Moreover, pictorial warnings led to lower intentions to serve sugary drinks to their child, feeling more in control of healthy eating decisions, greater thinking about the harms of sugary drinks, stronger negative emotional reactions, greater anticipated social interactions, lower perceived healthfulness of sugary drinks for their child, and greater injunctive norms to limit sugary drinks for their child (all *p* < 0.05). There was no evidence of difference between trial arms on noticing of the labels, appeal of sugary drinks, perceived amount of added sugar in sugary drinks, risk perceptions, or perceived tastiness of sugary drinks (all *p* > 0.05).

**Conclusions:**

Pictorial warnings reduced parents’ purchases of sugary drinks for their children in this naturalistic trial. Warnings on sugary drinks are a promising policy approach to reduce sugary drink purchasing in the US.

**Trial registration:**

The trial design, measures, power calculation, and analytic plan were registered before data collection at www.clinicaltrials.gov NCT04223687.

## Abstract 2

Using a naturalistic store laboratory, Marissa Hall and colleagues examine the impact of pictorial warnings on parents’ purchases of sugary drinks for their children.

## Author summary

**Why was this study done?:**

- Children in the US consume more than the recommended levels of sugary drinks, increasing their risk of a variety of chronic diseases, including type 2 diabetes and heart disease.
- Experimental studies of text-only sugary drink warning labels indicate that warnings reduce sugary drink consumption.
- To our knowledge, studies have not yet examined the effects of pictorial warnings on parents’ purchases of sugary drinks for their children or on purchases in naturalistic settings.

**What did the researchers do and find?:**

- Parents of children aged 2 to 12 years (n = 325) participated in a randomized trial. Participants were randomized to a pictorial warnings arm (sugary drinks displayed pictorial health warnings about type 2 diabetes and heart damage) or a control arm (sugary drinks displayed a barcode label). Participants completed a shopping task in a naturalistic store laboratory, where sugary drinks had the assigned label, and completed a survey.
- Exposure to pictorial warning labels on sugary drinks led to a 17% absolute reduction in the purchasing of sugary drinks compared to exposure to the barcode control label.

**What do these findings mean?:**

- Pictorial warnings are a promising option for reducing purchases of sugary drinks for children.
- Implementation of pictorial warning label policies could be an effective strategy for reducing sugary drink purchases and sugary drink–related health outcomes.
- Future studies should evaluate the long-term effects of warning labels on children’s sugary drink consumption and prevalence of diet-related chronic disease.

## Introduction

Consumption of beverages with added sugar (“sugary drinks”) remains well above recommended levels among both children and adults in the US, with 63% of children and 49% of adults consuming sugary drinks on a daily basis [1,2]. Sugary drink consumption contributes to numerous health problems including obesity, dental caries, type 2 diabetes, and heart disease [3–7]. Population-level strategies to lower sugary drink consumption are urgently needed. One promising approach to reducing sugary drink consumption is requiring health warnings to appear on sugary drinks. Since 2011, 7 US states have proposed legislation requiring that warnings stating the health consequences of sugary drinks appear on sugary drink containers, advertisements, or at the point of sale [8]. Globally, 7 countries have passed laws mandating “high in added sugar” warnings on products (including sugary drinks) that exceed thresholds for added sugar and other nutrients of concern such as sodium [9].

Meta-analyses of experimental studies have shown that sugary drink warnings reduce selection of sugary drinks [10–12]. Sugary drink warnings have also been shown to change possible psychological mediators of behavior change, including reductions in both perceived healthfulness of sugary drinks and intentions to consume sugary drinks [11]. However, most experiments to date have used artificial exposure to warnings via online surveys, with self-reported or hypothetical selection outcomes, and generalizability to real-stakes behavior in more realistic settings remains largely unknown [11]. To inform policy, experiments conducted in naturalistic settings are needed to better understand the impact of sugary drink warnings on consumer behavior.

Another open question is whether warnings with images depicting health harms (i.e., pictorial warnings) are effective in the context of sugary drinks. A large body of literature demonstrates that pictorial warnings on cigarette packs help people stop smoking and are more effective than text-only warnings [13–16]. Moreover, pictorial warnings may better reach vulnerable populations such as those with low socioeconomic status or low English proficiency [17,18]. Two previous online studies suggest that pictorial warnings lower intentions to purchase sugary drinks [17,19], and 1 quasi-experimental study found that pictorial warnings reduced purchases of sugary drinks relative to a no-label control, calorie labels, and text-only warning labels [20]. Although these initial findings are promising, only 1 other randomized controlled trial has evaluated the impact of pictorial warnings on sugary drink purchasing behavior [21].

This randomized controlled trial conducted in a naturalistic store laboratory aimed to examine whether pictorial health warnings on sugary drink containers lowered parents’ purchases of sugary drinks for their children [22]. We focused on parents, because parents exert strong influence over their children’s dietary intake and because the majority of children’s daily calories are consumed at home [23]. We also explored whether the impact of sugary drink warnings on purchasing behavior varied by participant characteristics. Finally, the study aimed to examine the impact of sugary drink warnings on secondary outcomes likely to predict longer-term behavior change, including label reactions, sugary drink attitudes, and behavioral intentions.

Study predictions came from health behavior theory [24–27], the Tobacco Warnings Model [28] (an empirical model of how pictorial cigarette warnings exert effects on behavior), as well as prior research on sugary drink warnings [10,29,30] and pictorial tobacco warnings [13,28,31]. We predicted that parents randomized to pictorial health warnings would be less likely to purchase a sugary drink for their child and would purchase fewer calories from sugary drinks, compared to parents randomized to exposure to a control label. We also predicted that participants randomized to pictorial warnings (versus a control label) would have stronger reactions to the labels and more negative attitudes and perceptions of sugary drinks.

## Methods

### Participants

From January to March 2020, study staff recruited participants from Central North Carolina through in-person recruitment, flyers, email listservs, Craigslist ads, Facebook ads, and word of mouth. Recruitment was completed in both English and Spanish. Due to COVID-19, the trial paused recruitment beginning in March 2020. After implementing a COVID-19 safety protocol, study staff resumed recruitment (using the same methods except in-person recruitment) in October 2020 and completed enrollment in March 2021 once the target sample size was met. Minor COVID-19–related modifications to the protocol are described below.

Interested individuals completed a screener to determine eligibility. To be eligible, participants had to be 18 years of age or older, the parent or guardian (hereafter “parent”) of at least 1 child between the ages of 2 and 12 who consumed one or more sugary drinks in the past week, able to read and speak English or Spanish, able to use a tablet or computer to take a survey, and able to attend 1 in-person study visit. Exclusion criteria included living in the same household as someone who already participated in the study or having had participated in a similar study in 2019 [22].

The University of North Carolina Institutional Review Board approved the study (IRB #19–0277). All study materials were available in English and Spanish. The trial design, measures, power calculation, and analytic plan were registered before data collection started at www.clinicaltrials.gov NCT04223687 (see **S1 Study Protocol and S1 Analytic Plan**). This study is reported as per the Consolidated Standards Of Reporting Trials (CONSORT) guideline (**S1 CONSORT Checklist**).

### Setting

The study took place at the UNC Mini Mart, a 245-square foot naturalistic store laboratory in Chapel Hill, North Carolina [22]. The UNC Mini Mart contains a commercial refrigerator, gondola shelving units, and a checkout stand with a point-of-sale system. Study staff stocked the UNC Mini Mart with 33 types of single-serve beverages, more than 130 types of food items, and 31 household good items.

To determine which single-serve beverages to stock, we used 2014 Nielsen Homescan Data [32] to examine the top selling beverages, by beverage category, at convenience stores in the US among all households with children aged 2 to 18, as well as all Hispanic households with children aged 2 to 18. Study staff stocked single-serve drinks in each of 6 beverage categories (fruit drinks/juices, sodas, milks, sports drinks, waters, and ready-to-drink teas; see **S1 Table** for a full list of beverages sold). For every sugary drink sold, there was a comparable nonsugary drink available (e.g., Ocean Spray Cranberry Juice Cocktail and Ocean Spray 100% Cranberry Juice). To better reflect the retail environment, the UNC Mini Mart also sold unflavored bottled water and noncalorically flavored sparkling water; these beverages did not have a corresponding sugary drink. Beverages were displayed in the refrigerator with each sugary drink placed side by side with its nonsugary drink equivalent. Prices for all beverages were similar to prices at local convenience stores. All sugary drinks and their nonsugary drink equivalents were sold for the same price, following the approach used in a prior study [30] (see **S1 Table** for prices).

To determine what food items and household goods to stock, research staff visited 5 local convenience stores and noted what types of food and household good products were available as well as the approximate distribution of the different types of products. We used this information, along with Euromonitor data [33], to identify top selling brands for different food and household good categories and to determine the final list of food and household goods to stock.

### Stimuli

To design the 2 pictorial warnings used in the trial, we implemented a multiphase warning development process [17]. First, we created text, icon, and pictorial warnings for 4 different possible health topics (added sugar, weight gain, heart damage, and type 2 diabetes); the topics were selected in consultation with legal experts and prioritized based on strength of the epidemiologic evidence and policy relevance. Then, a professional designer identified stock photographs representing each of the topics. After vetting an initial set of warning topics and photographs with a stakeholder advisory board (comprised of nutrition epidemiologists, a weight stigma expert, a public health lawyer, and leaders from local and national health organizations), we conducted 2 rounds of quantitative image pretesting using convenience samples of US adults recruited through Amazon Mechanical Turk (total *n =* 861).

For each warning topic, we selected the photograph that participants rated as most discouraging them from wanting to consume sugary drinks. We then created pictorial warnings that paired the warning statements with these photographs. Our designer also created icon warnings that paired the warning statements with icon versions of these photographs. We evaluated the warning topics (i.e., added sugar, weight gain, heart damage, and type 2 diabetes) and designs (i.e., text-only, icon, and pictorial) in an online experiment with 1,078 parents of children ages 2 to 12. The experiment found that all 4 warning topics performed similarly and that pictorial warnings outperformed both icon and text-only warnings [17]. Thus, for the current RCT, we opted to study pictorial warnings to maximize statistical power. Among the 4 warning topics, we excluded the “weight gain” warning depicting obesity out of concerns about promoting weight-related bias [34,35] and excluded the warning about added sugar because most US sugary drink warning proposals have focused on health outcomes [8]. The 2 final pictorial warnings used in the trial read “WARNING: Excess consumption of drinks with added sugar contributes to type 2 diabetes” and “WARNING: Excess consumption of drinks with added sugar contributes to heart damage.”

### Procedures

Individuals interested in the study could take the eligibility screener online or answer questions verbally while research staff screened for eligibility using the same online questionnaire. Before COVID-19, participants provided written informed consent at the study site; during COVID-19, participants provided verbal consent by phone prior to the study visit. To mask the purpose of the study, all study materials described the purpose of the research study as being to understand the factors that affect consumers’ purchasing decisions in a convenience store environment.

This study used a parallel arm study design. When participants arrived at the study appointment, study staff assigned them to 1 of the 2 trial arms: pictorial warnings or control labels. Study staff consulted a prepopulated list of allocations and assigned the participant the next allocation on the list. The study project manager list was created using an Excel function with a 1:1 simple allocation ratio prior to the study’s start.

**Fig 1:**
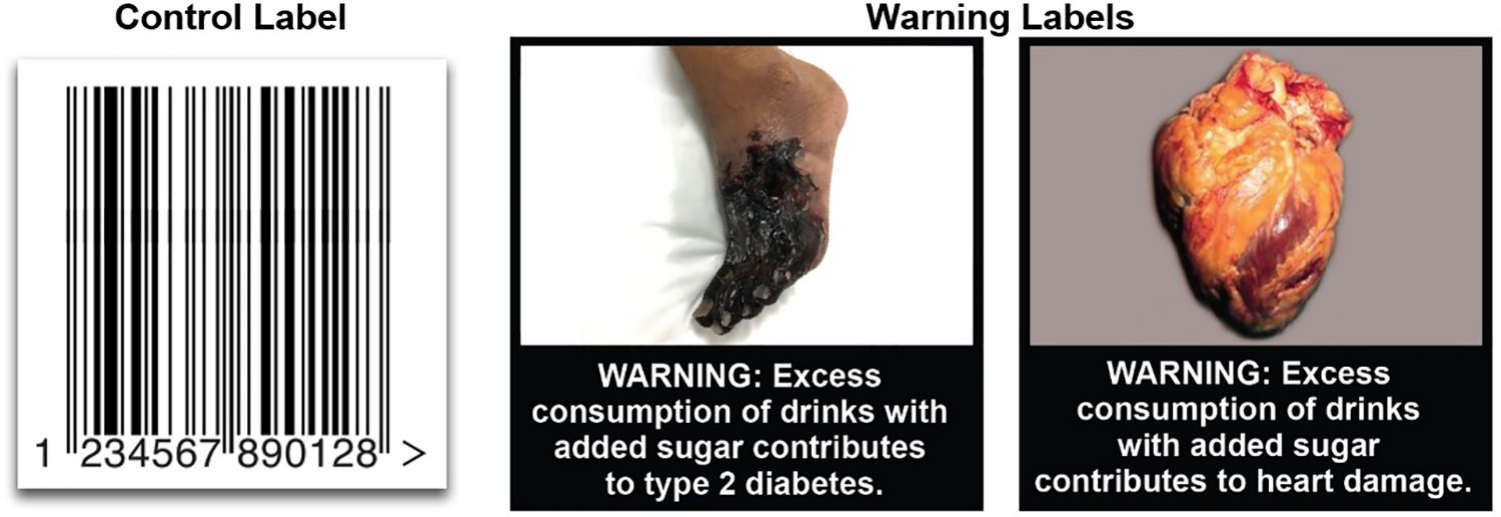
Study stimuli in a trial evaluating pictorial health warnings for sugary drinks.

**Fig 2:**
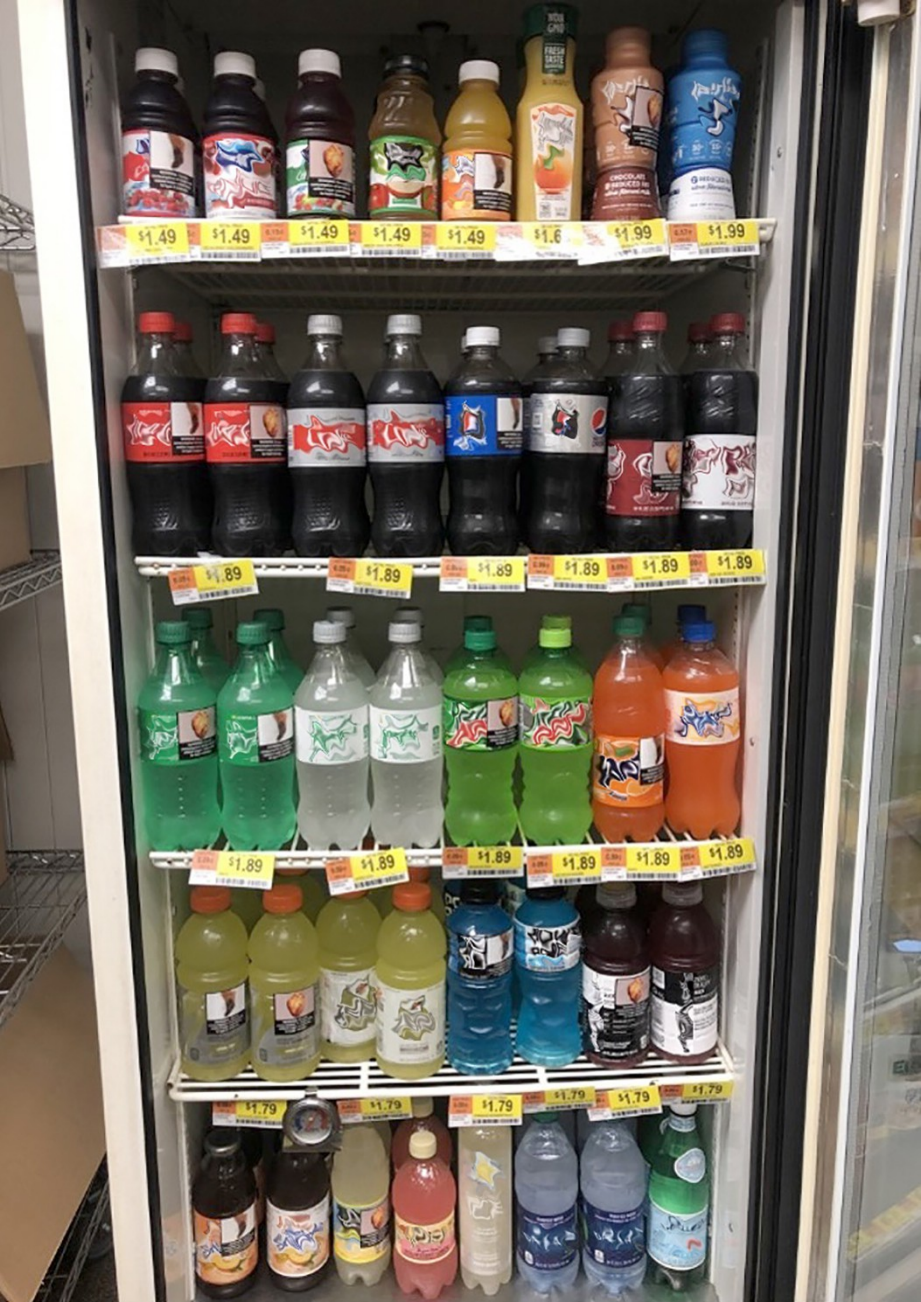
Photographs of UNC Mini Mart during a trial evaluating graphic health warnings for sugary drinks.

Research staff prepared the UNC Mini Mart before participants’ arrival based on the participant’s assigned trial arm (i.e., pictorial warning arm or control arm). In the pictorial warning arm, research staff applied one of 2 warning labels (**Fig 1**) to the front of all sugary drink containers in the UNC Mini Mart. Approximately half of the sugary drinks displayed the heart damage warning label, and the other half displayed the type 2 diabetes label. In the control arm, research staff applied a control label to all sugary drinks. As in prior studies [30,36], we selected a barcode image for the control label because it is a neutral image that already appears elsewhere on beverage containers. Using a neutral label, rather than a no-label control, ensured that the study controlled for the effect of putting a new label on sugary drinks and for the amount of branding obscured by the labels. Both pictorial warnings as well as the control labels measured 1.85 inches × 1.85 inches in size (see **Fig 2** for images of the labeled drinks in the store).

Before entering the UNC Mini Mart, research staff instructed participants to buy 1 snack and 1 beverage for their child between the ages of 2 and 12, as well as 1 household item. If a participant had more than 1 child between the ages of 2 and 12 that consumed sugary drinks, the research staff randomly selected one of the participants’ children for whom the participant would shop. The 3-item shopping task was designed to mask the purpose of the study. Research staff informed the participants that one of the items would be randomly selected at the checkout counter for the participant to take home, and the cost of the item would be subtracted from their $40 incentive. However, unbeknownst to the participants, the randomizer was set up to always select the beverage in order to simplify inventory management. Research staff waited inside (before COVID-19) or outside (during COVID-19) the UNC Mini Mart while the participant completed the shopping task, then checked out the participant at the checkout counter and handed the participant their child’s beverage. The point-of-sale system automatically recorded participants’ purchases. These electronic purchase records were verified against study staff’s written documentation of purchases.

After the shopping task, participants completed a survey programmed in Qualtrics on a computer or tablet in a separate room. Upon completing the survey, the research staff provided the participant with their cash incentive and a 1-page informational handout about sugary drinks. To simplify incentive distribution, research staff rounded down the cost of the beverage to the nearest dollar ($1 or $2, depending on beverage), so all participants left the study with their selected beverage and either $38 or $39.

### Measures

The primary trial outcome was whether participants selected a sugary drink for their child. A secondary purchase outcome was total calories purchased from sugary drinks. Secondary psychosocial outcomes, measured in the survey, included reactions to the trial labels, sugary drink attitudes, and behavioral intentions. Exact survey items, response options, and sources of measures appear in **S2 Table**. All psychosocial outcomes were assessed using Likert-style response scales ranging from 1 to 5, except where noted. Cronbach’s alpha was greater than 0.70 for all scales. The survey assessed label reactions, including whether participants noticed the labels in the UNC Mini Mart (response options were yes/no), whether the labels made them feel more in control of making healthy eating decisions (response options were yes/no), how much the label(s) made them think about the health problems caused by consuming sugary drinks, how much the label(s) made them experience negative emotional reactions (anxious, scared, guilty), and their likelihood of discussing the label(s) with others (i.e., anticipated social interactions).

The survey assessed several variables representing sugary drink attitudes and intentions. Participants responded to questions about 6 types of sugary drinks (regular soda or soft drinks, regular sports drinks, regular flavored waters, fruit-flavored drinks [not 100% juice], sweetened prepackaged teas, and flavored milks). For each type of sugary drink, the survey displayed images of example drinks with the label corresponding to the participant’s condition (warning label or barcode). The outcomes measured included intentions about frequency of serving sugary drinks to one’s child in the next week (response options ranged from intentions to serve sugary drinks 0 times per week to 21 times per week), perceived amount of added sugar in sugary drinks, perceived healthfulness of sugary drinks for one’s child, appeal of sugary drinks for one’s child, and perceived tastiness of sugary drinks to one’s child. The survey also assessed perceived likelihood of sugary drinks increasing their child’s risk of heart damage, type 2 diabetes, and health problems, as well as injunctive norms to limit sugary drinks for their child.

Finally, the survey assessed participants’ beliefs about the purpose of the study with an open-ended question presented before any other items. Researchers coded responses to this item to determine whether participants correctly guessed the purpose of the study (i.e., to assess the impact of warning labels on drink purchasing).

### Analyses

We powered the study assuming warning labels would exert a small-to-medium effect size (Cohen’s *d* = 0.32) on our primary outcome, the effect observed in a study using similar methods and stimuli [30]. Using these specifications and a 2-sided alpha of 0.05, we determined a necessary sample of 314 to detect an effect of *d* = 0.32 or larger with 80% power. To account for potential missing data or dropout occurring during study visits, we aimed to enroll 326 participants (163 in each arm), as described in our prespecified analytic plan.

Analyses used a critical alpha of 0.05 and were conducted in Stata/SE version 16. Although the preregistration specified that we would examine whether randomization created equivalent demographic groups, we did not perform these analyses to ensure that we aligned with CONSORT guidance [37] and the guidance of a peer reviewer with statistical expertise.

For hypothesis testing, we derived *p*-values and 95% confidence intervals using independent z-tests comparing proportions for dichotomous outcomes and independent samples *t* tests for all Likert-style items and total calories from sugary drinks. To put the effects into a common metric, we calculated a standardized effect size (Cohen’s *d*) [38]. Sensitivity analyses (not preregistered but included based on peer reviewer feedback) excluding participants who correctly identified the purpose of the study (*n =* 4) revealed results for the primary outcome of purchasing a sugary drink that were identical in direction of effect and statistical significance, so subsequent analyses included all participants.

We explored whether participant and child characteristics moderated the impact of warning labels on the primary outcome of selecting a sugary drink by including an interaction term between study arm and each characteristic in logistic regression models. These characteristics included parents’ age, gender, sexual orientation, race/ethnicity, educational attainment, annual household income, Nutrition Facts Panel use, frequency of needing help reading medical information, language of survey administration, age of the child the parent shopped for, gender of the child the parent shopped for, the child’s consumption of sugary drinks, and whether the parent participated before or during COVID-19. These exploratory moderation analyses corrected for multiple comparisons using the Holm–Bonferroni method [39]. To test these interactions, we ran separate logistic regression models for each moderator, regressing trial arm, the moderator, and their interaction on the primary outcome and examined the statistical significance of the moderator term.

## Results

**Fig 3:**
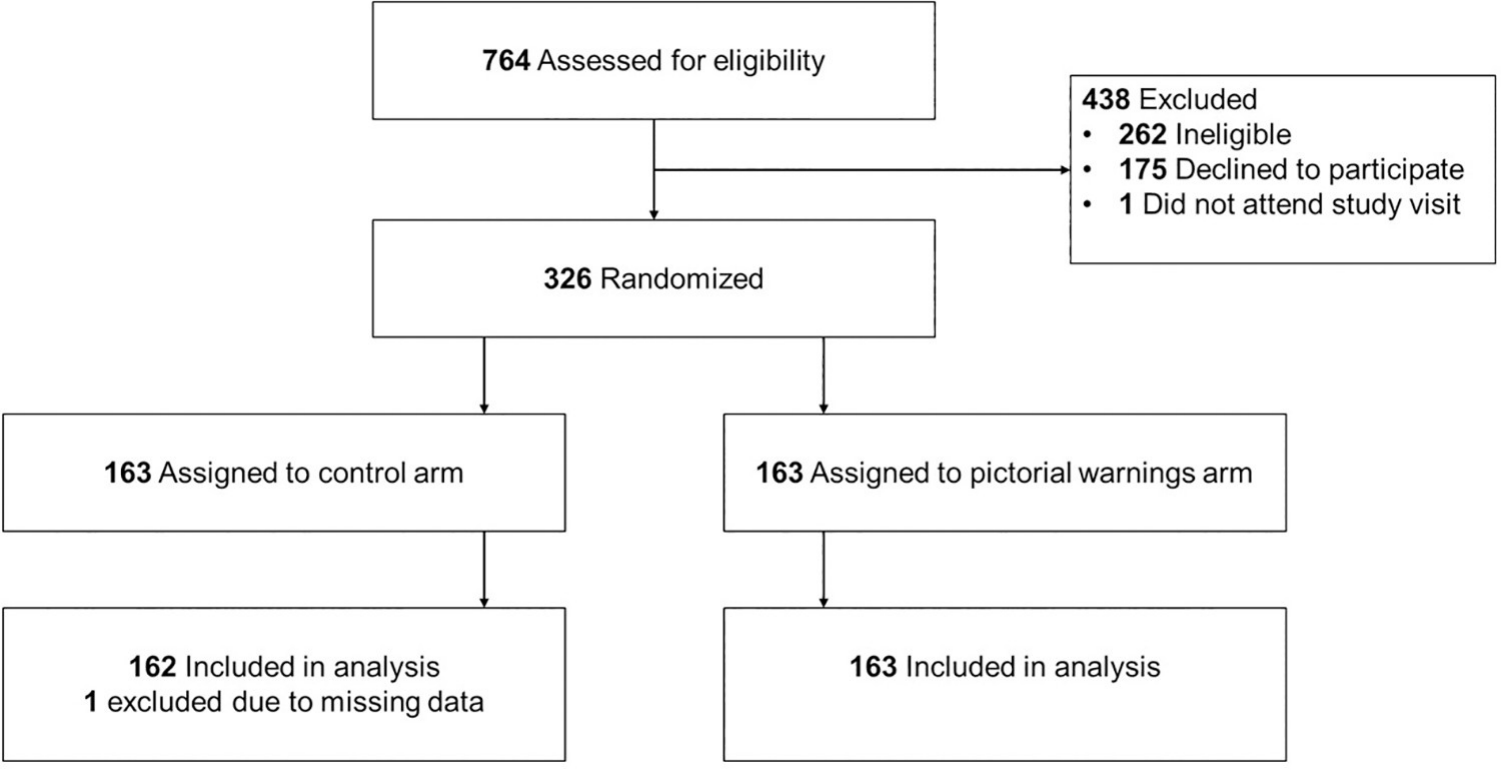
CONSORT flow diagram.

A total of 326 parents enrolled in the study; we included 325 participants in analyses, excluding 1 participant who had missing data on the primary outcome due to a discrepancy between the point-of-sale record and the research staff’s record (see **Fig 3** for CONSORT diagram). Of the 325 participants included in analyses, 162 were randomized to the control arm, and 163 were randomized to the pictorial warnings arm. Parents’ mean age was 38 years, and 77% identified as women (**Table 1**). Slightly less than half (45%) identified as non-Hispanic white, 25% identified as non-Hispanic Black or African American, and 20% identified as Hispanic. About half (55%) of parents had an annual household income of less than $50,000, and 42% had a high school diploma or less. About a third (38%) of parents shopped for a child between the ages of 2 and 5 years, and 62% shopped for a child between 6 and 12 years.

**Table 1:** Participant characteristics (*n =* 325).

Characteristic	Control arm		Pictorial warnings arm	
	***n***	**%**	***n***	**%**
**Age, in years**				
18–29	24	15%	21	13%
30–39	65	40%	74	45%
40–49	53	33%	54	33%
50+	20	12%	14	9%
Mean (SD)	38.9	8.3	37.8	7.8
**Gender**				
Man	41	25%	32	20%
Woman	120	74%	130	80%
Another gender identity	1	1%	1	1%
**Sexual orientation**				
Straight or heterosexual	148	94%	145	90%
Gay, lesbian, bisexual, or homosexual	6	4%	14	9%
Another sexual orientation	4	3%	3	2%
**Race and ethnicity**				
Non-Hispanic white	72	46%	70	44%
Hispanic white	9	6%	10	6%
Hispanic, no race reported	14	9%	15	9%
Hispanic, other race(s)	8	5%	9	6%
Non-Hispanic Black or African American	46	29%	34	21%
Non-Hispanic Asian	6	4%	7	4%
Non-Hispanic, more than 1 race	3	2%	13	8%
Non-Hispanic, other race	0	0%	2	1%
**Educational attainment**				
Less than high school diploma or GED	11	7%	15	9%
High school diploma or GED	55	35%	55	34%
Four-year college degree	42	27%	46	29%
Master’s degree, graduate degree, or more	47	30%	44	28%
**Annual household income**				
$0–$24,999	49	30%	50	32%
$25,000–$49,999	39	24%	41	26%
$50,000–$74,999	16	10%	18	11%
$75,000+	58	36%	49	31%
**Number of people in household, mean (SD)**	3.6	1.2	3.6	1.3
**Body mass index (kg/m^2^)**				
<18.5	6	4%	4	3%
18.5 to <25	43	29%	43	28%
25 to <30	43	29%	45	30%
≥30	57	38%	60	39%
Mean (SD)	29.7	9.9	29.3	8.0
**Nutrition Facts Panel use**				
Never or rarely	26	16%	25	16%
Sometimes	46	29%	49	30%
Often or all the time	89	55%	87	54%
**Frequency of needing help reading medical information**				
Never	130	81%	132	81%
Sometimes	23	14%	18	11%
Often/Always	7	4%	12	7%
**Language of survey administration**				
English	142	88%	140	86%
Spanish	20	12%	23	14%
**Reads and speaks. . .**				
Mostly or only English	132	81%	130	80%
Spanish and English equally	10	6%	14	9%
Mostly or only Spanish	20	12%	19	12%
**Age of child the parent shopped for, in years**				
2–5	61	38%	63	39%
6–12	101	62%	100	61%
Mean (SD)	7.3	3.4	7.1	3.3
**Gender of child the parent shopped for**				
Boy	72	44%	75	46%
Girl	88	54%	88	54%
Another gender identity	2	1%		
**Child consumed sugary drink 1/wk or more over past 30 days (not mutually exclusive)**				
Soda	68	42%	58	36%
Sports drinks	50	31%	50	31%
Flavored water	43	27%	38	24%
Fruit drink	102	64%	95	59%
Flavored milk	102	65%	98	61%
Sweetened coffee or tea	41	26%	35	22%
**Time of participation**				
Pre-COVID-19 pandemic	64	40%	65	40%
During COVID-19 pandemic	98	60%	98	60%

GED, General Educational Diploma; SD, standard deviation.

Missing demographic data ranged from 0% to 7%.

Exposure to pictorial warnings on sugary drinks lowered parents’ likelihood of buying a sugary drink for their child, leading to a 17-percentage point reduction in purchasing sugary drinks (95% confidence interval for difference: 7% to 27%). Among parents in the pictorial warnings arm, 28% bought a sugary drink for their child, compared to 45% of parents who viewed a control label (*p* = 0.002). Parents in the pictorial warnings arm also purchased fewer calories from sugary drinks compared to those in the control arm (52 kcal in pictorial warnings arm versus 82 in control arm, *d* = −.33, *p* = 0.003; **Table 2**). The effect of pictorial warnings on parents’ selection of a sugary drink for their child did not differ by any of the 13 participant characteristics examined, including age (*p* = 0.507), gender (*p* = 0.033), sexual orientation (*p* = 0.599), race/ethnicity (*p* = 0.045), educational attainment (*p* = 0.863), annual household income (*p* = 0.916), Nutrition Facts Panel use (*p* = 0.718), health literacy (*p* = 0.852), language of survey administration (*p* = 0.389), age of the child the parent shopped for (*p* = 0.559), gender of the child the parent shopped for (*p* = 0.630), child’s consumption of sugary drinks (*p* = 0.760), and whether the participant participated before or during COVID-19 (*p* = 0.537); no interactions were statistically significant after Holm–Bonferroni correction; see **S3 Table** for effects by subgroup.

**Table 2:** Impact of pictorial warnings on sugary drink purchases and secondary outcomes.

Outcome	Control arm		Pictorial warnings arm				
	**n or mean**	**% or (SD)**	**n or mean**	**% or (SD)**	**Difference** **(95% CI)**	***p***	**Cohen’s *d***
**Purchase outcomes**							
Purchased a sugary drink (primary outcome)	73	45%	46	28%	−17% (−27%, −7%)	0.002	−0.41
Total calories from sugary drinks (in kcal)	82.1	(97.2)	51.7	(86.8)	−30.4 (−50.5, −10.3)	0.003	−0.33
**Label reactions**							
Noticed label	76	47%	84	52%	5% (−6%, 16%)	0.373	0.11
Felt more in control of healthy eating decisions	48	30%	118	73%	43% (34%, 53%)	<0.001	1.02
Thinking about harms of drinking sugary drinks	1.90	(1.25)	4.21	(0.95)	2.31 (2.07, 2.55)	<0.001	2.08
Negative emotional reactions	1.51	(0.82)	3.38	(1.12)	1.87 (1.66, 2.09)	<0.001	1.91
Anticipated social interactions	2.16	(1.32)	3.89	(1.24)	1.73 (1.45, 2.01)	<0.001	1.35
Perceived amount of added sugar in sugary drinks	3.99	(0.67)	4.08	(0.77)	0.10 (−0.06, 0.26)	0.220	0.14
**Sugary drink attitudes and intentions**							
Perceived healthfulness of sugary drinks for child	2.24	(0.80)	1.98	(0.82)	−0.26 (−0.44, −0.08)	0.004	−0.32
Appeal of sugary drinks for child	3.32	(0.96)	3.24	(0.98)	−0.08 (−0.30, 0.13)	0.439	−0.09
Perceived tastiness of sugary drinks for child	3.47	(0.89)	3.30	(0.95)	−0.17 (−0.37, 0.03)	0.104	−0.18
Perceived likelihood of child experiencing health problems due to sugary drinks	3.95	(0.99)	4.07	(0.92)	0.12 (−0.10, 0.33)	0.288	0.12
Injunctive norms to limiting sugary drinks for child	3.21	(1.33)	3.6	(1.20)	0.39 (0.11, 0.67)	0.006	0.31
Intentions to give sugary drinks to child	1.26	(1.53)	0.91	(1.15)	−0.35 (−0.64, −0.05)	0.022	−0.26

*p* for binary outcomes from independent-samples z-tests. *p* for continuous outcomes from independent-samples *t* tests. Missing data ranged from 0.4% to 5%.

Nearly three-quarters (73%) of parents in the pictorial warnings arm reported the label made them feel more in control of healthy eating decisions, compared with 30% in the control arm (*p* < 0.001; **Table 2**). Exposure to pictorial warnings on sugary drinks also led to stronger label reactions, including greater thinking about the harms of sugary drinks (*d* = 2.08, *p* < 0.001), greater negative emotional reactions (*d* = 1.91, *p* < 0.001), and greater anticipated social interactions (*d* = 1.35, *p* < 0.001). Pictorial warnings changed several sugary drink attitudes and intentions, including lower perceived healthfulness of sugary drinks for their child (*d* = −0.32, *p* = 0.004), greater injunctive norms to limit sugary drinks for their child (*d* = 0.31, *p* = 0.006), and lower intentions to serve sugary drinks to their child (*d* = −0.26, *p* = 0.022). Pictorial warnings did not lead to differences in noticing of the label (*p* = 0.373), appeal of sugary drinks for child (*p* = 0.439), perceived amount of added sugar in sugary drinks (*p* = 0.220), perceived tastiness of sugary drinks to their child (*p* = 0.104), or perceived likelihood of child experiencing health problems due to sugary drinks (*p* = 0.288).

## Discussion

In this randomized controlled trial in a naturalistic store laboratory, exposure to 2 pictorial warnings (one about heart damage and one about type 2 diabetes) reduced parents’ likelihood of selecting sugary drinks for their children, with no differences in impacts by participant characteristics. The pictorial warnings also reduced calories purchased from sugary drinks. As assessed in the post-shopping survey, pictorial warnings led to lower intentions to serve sugary drinks to their child, feeling more in control of healthy eating decisions, greater thinking about the harms of sugary drinks, stronger negative emotional reactions, greater anticipated social interactions, lower perceived healthfulness of sugary drinks for their child, and greater injunctive norms to limit sugary drinks for their child.

Pictorial warnings reduced parents’ selection of sugary drinks for their children by 17 percentage points, from 45% in the control arm to 28% in the warnings arm. This change represents a relative reduction of more than 30%. Pictorial warnings also led to an approximately 37% relative reduction in calories purchased from sugary drinks (82 kcal in the control group versus 52 kcal in the pictorial warnings arm). This relative reduction is slightly larger than the approximately 22% relative reduction in a prior trial of text-only warnings using similar methods [30]. The current trial is in line with meta-analyses that have found that sugary drink warnings reduce selection of sugary drinks [10–12]. The observed reduction in sugary drink purchases and calories from sugary drinks could yield meaningful health benefits at the population level. One simulation modeling study found that if sugary drink warnings reduced caloric intake by 31 kcal per day, obesity prevalence in the US would decline by 3.1 percentage points in adults [40].

The current study, one of the first randomized controlled trials to examine the impact of *pictorial* warnings on purchasing behavior in a real-world setting, suggests that pictorial warnings are a promising policy option for reducing parents’ sugary drink purchases for children. Prior studies have similarly found that pictorial warnings are perceived as more effective than text-only warnings in the context of foods [41] and sugary drinks [17,19]. A quasi-experiment found that pictorial warnings displayed on sugary drink dispensers were associated with reduced purchases of sugary drinks in a real-world cafeteria setting [20]. These findings stand in contrast with a study finding that pictorial warnings on sugary drinks did not affect purchasing compared to a control [21]. The differences in findings between our study and that prior study could be because the prior study placed labels on the side of drinks rather than the front, measured drink selection among adults for themselves rather than for their children, and enrolled participants who may not have been sugary drink consumers, potentially attenuating treatment effects. Future longitudinal studies, especially those conducted in real-world settings such as actual grocery stores, will help to establish the generalizability of the observed effects, as well as whether warnings’ effects are sustained over time.

This trial found that the benefits of pictorial warnings on purchasing behavior did not differ by any of the 13 participant characteristics explored, including income, education, race/ethnicity, time of data collection (before versus during COVID-19), and children’s frequency of sugary drink consumption. Although this study may not have been powered to detect small differences in warnings’ impacts between groups, the lack of moderation by demographic characteristics observed here is in line with prior research demonstrating that sugary drink warnings generally appear to benefit diverse groups similarly and therefore may be unlikely to exacerbate diet-related disparities [30,36,42–45].

In addition to changing parents’ purchasing behavior, pictorial warnings changed several outcomes that are precursors to longer-term behavior change. The Tobacco Warnings Model posits that health warnings change behavior by eliciting attention, increasing negative emotional reactions, making people think about the harms of consuming the product, sparking social interactions, and ultimately lowering intentions to purchase or consume the product [28]. Our results support the application of the Tobacco Warnings Model in the context of sugary drink warnings: In our study, pictorial warnings led to greater thinking about harms of sugary drinks, negative emotional reactions, and anticipated social interactions, while leading to lower intentions to serve sugary drinks to children. Our findings are in line with a prior experimental study of pictorial sugary drink warnings, which found that the impact of warnings on increasing intentions to purchase water instead of sugary drinks was mediated through negative affect and greater consideration of the health effects of sugary drinks [20].

In our study, pictorial warnings changed some attitudes and perceptions (e.g., perceived healthfulness of sugary drinks), but not others (e.g., perceived risk of the harms of sugary drinks). The null finding for perceived risk is in line with meta-analyses of pictorial warnings on cigarette packs, which find that pictorial warnings do not change smoking-related risk beliefs compared to text-only warnings [13,46]. However, a meta-analysis of sugary drink warnings found that sugary drink warnings led to greater risk perceptions than controls [11]. Additional research would be useful to continue to identify the psychological mechanisms through which sugary drink warnings influence behavior change [47], as well as to examine whether the impact of pictorial warnings differs by sugary drink category in light of prior research showing differential effects of text-only warnings by category [48].

Taken together, existing research points toward pictorial warnings as being more effective than both controls and text-only warnings. No jurisdictions currently require pictorial warnings for sugary drinks; over 100 countries, however, require pictorial warnings for cigarette packs [49]. Pictorial sugary drink warning policies could be a useful complement to nutrient warning policies. The inclusion of imagery in warnings may also help to communicate risk to lower literacy or non-English-speaking populations, in light of research showing a stronger effect of icon warnings versus text-only warnings among parents self-identifying as Hispanic with limited English proficiency [17]. Moreover, pictorial elements could be incorporated into other communications approaches, such as sugary drink reduction social media campaigns or point-of-sale signage.

Careful attention should be given to ensuring that pictorial warnings do not heighten weight-related bias, in light of a prior study finding that pictorial warnings depicting obesity, type 2 diabetes, and tooth decay led to greater weight bias than a control label [34]. Moreover, additional research is warranted to understand the potential impact of warning labels on people with disordered eating behavior, as 1 study found that menu calorie labels may negatively impact people with a diagnosed eating disorder [50]. When designing and pretesting warning labels, researchers could consider vetting labels with experts on weight-related bias and disordered eating; gathering quantitative data on stigma, weight bias, or self-esteem; and collecting qualitative data about potential unintended consequences of warnings.

Strengths of this study include the use of professionally designed stimuli developed using a multiphase process, the objective measurement of a behavioral outcome, and the inclusion of a diverse sample of parents with respect to race/ethnicity and socioeconomic status. Additionally, the trial took place in a naturalistic store laboratory in which participants were incentivized to choose products they wanted to take home. One limitation is the brief exposure to stimuli during 1 study visit. Future longitudinal studies will shed light on the extent to which warnings’ impacts are sustained over time. Another limitation was that we were unable to compare pictorial warnings to text-only warnings, so the added benefit of pictorial elements above and beyond text warnings in this context remains unknown. Additionally, the trial evaluated only 2 warnings, each about a single health problem; the impact of other health topics or using multiple health topics on 1 warning label remains to be established. In the Mini Mart, sugary drinks and nonsugary drinks were presented side by side, as is typical in convenience store settings. This presentation may have made substitution to nonsugary drinks more likely than would be the case in grocery store settings where sugary drinks and nonsugary drink options are not always side by side or in the same section of the store. Future studies could evaluate the impact of warnings in different store types such as grocery store settings. Finally, participants may have inferred the purpose of the study and changed their behavior accordingly. However, both groups received the same shopping instructions and researchers attempted to mask the purpose of the study in all recruitment and study materials and by having parents select a food item and household good in addition to a drink. These efforts appear to have succeeded given that only 4 participants correctly guessed the purpose of the study, and the primary outcome results did not change when excluding these participants.

## Conclusions

Findings from this trial in a naturalistic setting suggest that pictorial warnings on sugary drinks could be an effective approach for reducing parents’ purchases of sugary drinks for their children. The warnings worked similarly well across all demographic groups examined. Taken together with prior research, this study suggests that a policy to require sugary drink warnings that include pictorial elements could reduce sugary drink purchases.

## Supporting information

S1 CONSORT Checklist CONSORT 2010 checklist of information to include when reporting a randomized trial. (DOC)

S1 Table Beverage stocked in the UNC Mini Mart during a trial evaluating pictorial health warnings for sugary drinks. (DOCX)

S2 Table Survey items and response options used in posttest survey in a trial evaluating pictorial health warnings for sugary drinks. (DOCX)

S3 Table Percent of parents selecting sugary drinks by trial arm. **Interaction *p*-values from logistic regression models.** No *p*-values were statistically significant after applying Holm–Bonferroni correction. *p from Wald test for joint interaction.
(DOCX)

S1 Study Protocol Protocol for a study examining the impact of warnings on sugar-sweetened beverages. (PDF)

S1 Analytic Plan Impact of warnings on sugar-sweetened beverages, hypotheses, and analytic plan. (PDF)

S1 Data Public dataset. (DTA)

S1 Do File Public Do file. (DO)
