# Supplementary material for: The impact of pictorial health warnings on purchases of sugary drinks for children: A randomized controlled trial
Source: PLoS Med. 2022 Feb 1;19(2):e1003885. doi: 10.1371/journal.pmed.1003885 (PMC8806063; doi:10.1371/journal.pmed.1003885)
Supplement: S1 Table — (DOCX) [file pmed.1003885.s002.docx]

**S1 Table**. **Beverage stocked in the UNC Mini Mart during a trial evaluating pictorial health warnings for sugary drinks.**

| **Beverage category** | **Beverage name** | **Sugary drink** | **Size of beverage (mL)** | **Price** |
| --- | --- | --- | --- | --- |
| Soda | Coca Cola | Yes | 591mL | $1.89 |
| Soda | Diet Coke | No | 591mL | $1.89 |
| Soda | Pepsi | Yes | 591mL | $1.89 |
| Soda | Diet Pepsi | No | 591mL | $1.89 |
| Soda | Dr. Pepper | Yes | 591mL | $1.89 |
| Soda | Diet Dr. Pepper | No | 591mL | $1.89 |
| Soda | Sprite | Yes | 591mL | $1.89 |
| Soda | Sprite Zero | No | 591mL | $1.89 |
| Soda | Mountain Dew | Yes | 591mL | $1.89 |
| Soda | Diet Mountain Dew | No | 591mL | $1.89 |
| Soda | Fanta | Yes | 591mL | $1.89 |
| Soda | Diet Sunkist | No | 591mL | $1.89 |
| Sports drink | Gatorade Lemon-Lime | Yes | 591mL | $1.79 |
| Sports drink | Gatorade Zero Lemon-Lime | No | 591mL | $1.79 |
| Sports drink | Powerade Mtn Berry Blast | Yes | 591mL | $1.79 |
| Sports drink | Powerade Zero Mtn Berry Blast | No | 591mL | $1.79 |
| Tea | Snapple Peach Tea | Yes | 473mL | $1.19 |
| Tea | Diet Snapple Peach Tea | No | 473mL | $1.19 |
| Fruit drink/juice | Minute Maid Lemonade | Yes | 500mL | $1.49 |
| Fruit drink/juice (before COVID) | Minute Maid Light Lemonade | No | 500mL | $1.49 |
| Fruit drink/juice (after COVID) | Nature’s Twist Sugar Free  Strawberry Lemonade | No | 473mL | $1.49 |
| Fruit drink/juice | Ocean Spray Cranberry Juice Cocktail | Yes | 295mL | $1.49 |
| Fruit drink/juice | Ocean Spray 100% cranberry juice | No | 295mL | $1.49 |
| Fruit drink/juice | Ocean Spray Cran-Apple | Yes | 295mL | $1.49 |
| Fruit drink/juice | Minute Maid 100% Apple Juice | No | 295mL | $1.49 |
| Fruit drink/juice | Welch's Orange Pineapple | Yes | 295mL | $1.49 |
| Fruit drink/juice | Simply 100% Orange Juice | No | 340mL | $1.69 |
| Milk | Fairlife 2% chocolate milk | Yes | 340mL | $1.99 |
| Milk | Fairlife 2% milk | No | 340mL | $1.99 |
| Water | Vitamin Water XXX | Yes | 591mL | $1.79 |
| Water | Vitamin Water Zero XXX | No | 591mL | $1.79 |
| Water | Sparkling Ice Lemonade | No | 502.8mL | $1.39 |
| Water | Dasani | No | 591mL | $1.69 |
| Water | San Pellegrino | No | 500mL | $1.49 |
