# Supplementary material for: The impact of pictorial health warnings on purchases of sugary drinks for children: A randomized controlled trial
Source: PLoS Med. 2022 Feb 1;19(2):e1003885. doi: 10.1371/journal.pmed.1003885 (PMC8806063; doi:10.1371/journal.pmed.1003885)
Supplement: S2 Table — (DOCX) [file pmed.1003885.s003.docx]

**S2 Table**. **Survey items and response options used in post-test survey in a trial evaluating pictorial health warnings for sugary drinks.**

| A30  prompt_1 | [page break]  **Please read each question carefully. You will not be able to change your answers after you go to the next page.** |  |
| --- | --- | --- |
| ntcng_e  Noticing – Intervention Arm**  (Adapted from  Roberto et al., 2016 [1] and VanEpps & Roberto, 2016 [2]) | **[Only show for Intervention Version]**  Some of the beverages in the store may have had extra picture warning labels (stickers) added on top of the regular packaging, like in these pictures above. Did you see these labels?  **[Show picture of generic soda bottle with warning labels located where we placed labels in the store].**  **[page break]** | 0= No  1= Yes |
| ntcng_c  Noticing – Control Arm**  (Adapted from  Roberto et al., 2016 [1] and VanEpps & Roberto, 2016 [2]) | **[Only show for Control Version]**  Some of the beverages in the store may have had extra white rectangle labels (stickers) added on top of the regular packaging, like in this picture above. Did you see these labels?  **[Show picture of generic soda bottle with barcode label located where we placed labels in the store].**  **[page break]** | 0 = No  1= Yes |
|  | **H: Knowledge/Perceptions of added sugar - self** |  |
| H10  prompt_4 | The next questions are about different types of beverages.  [Show image with warning label or barcode, based on trial arm] |  |
| H30  know_soda  Knowledge of added sugar (SSBs)  (Adapted from  Roberto et al., 2016 [1] and VanEpps & Roberto, 2016 [2]) | How much **added sugar** do you think is in **regular (non-diet) soda and soft drinks** like the ones above?  **[show image of regular sodas –Pepsi, Sprite]** | 1 (None)  2  3  4  5 (A lot) |
| H40  know_dtsoda  Knowledge of added sugar (non-SSBs)  (Adapted from  Roberto et al., 2016 [1] and VanEpps & Roberto, 2016 [2]) | How much **added sugar** do you think is in **diet soda and soft drinks** like the ones above?  **[show image of diet sodas – Diet Pepsi, Sprite Zero]** | 1 (None)  2  3  4  5 (A lot) |
| H70  know_sprt  Knowledge of added sugar (SSBs)  (Adapted from  Roberto et al., 2016 [1] and VanEpps & Roberto, 2016 [2]) | How much **added sugar** do you think is in **regular (non-diet) sports drinks** like the ones above?  **[show image of regular Gatorade]** | 1 (None)  2  3  4  5 (A lot) |
| H80  know_dtsprt  Knowledge of added sugar (non-SSBs)  (Adapted from  Roberto et al., 2016 [1] and VanEpps & Roberto, 2016 [2]) | How much **added** **sugar** do you think is in **diet sports drinks** like the ones above?    **[show image of Gatorade zero]** | 1 (None)  2  3  4  5 (A lot) |
| H70  know_flvwtr  Knowledge of added sugar (SSBs)  (Adapted from  Roberto et al., 2016 [1] and VanEpps & Roberto, 2016 [2]) | How much **added sugar** do you think is in **regular (non-diet) flavored waters** like the ones above?  **[show image of regular Vitamin Water]** | 1 (None)  2  3  4  5 (A lot) |
| H80  know_dtflvwtr  Knowledge of added sugar (non-SSBs)  (Adapted from  Roberto et al., 2016 [1] and VanEpps & Roberto, 2016 [2]) | How much **added** **sugar** do you think is in **diet flavored waters** like the ones above?    **[show image of Vitamin Water Zero]** | 1 (None)  2  3  4  5 (A lot) |
| H90  know_wtr  Knowledge of added sugar (non-SSBs)  (Adapted from  Roberto et al., 2016 [1] and VanEpps & Roberto, 2016 [2]) | How much **added sugar** do you think is in **bottled waters** like the ones above?  **[show image of bottled waters (still + sparkling) as used in store]** | 1 (None)  2  3  4  5 (A lot) |
| H110  know_frtflv  Knowledge of added sugar (SSBs)  (Adapted from  Roberto et al., 2016 [1] and VanEpps & Roberto, 2016 [2]) | How much **added** **sugar** do you think is in **fruit-flavored drinks** **(not 100% juice)** like the ones above?  **[show image of fruit drinks – Sunny D Orange Drink, Ocean Spray Cranberry Juice Cocktail]** | 1 (None)  2  3  4  5 (A lot) |
| H100  know_frt  Knowledge of added sugar (non-SSBs)  (Adapted from  Roberto et al., 2016 [1] and VanEpps & Roberto, 2016 [2]) | How much **added sugar** do you think is in **100% fruit juices** like the ones above?  **[show image of 100% fruit juices – Tropicana 100% Orange Juice, Ocean Spray 100% Cranberry Juice]** | 1 (None)  2  3  4  5 (A lot) |
| H110  know_swttea  Knowledge of added sugar (SSBs)  (Adapted from  Roberto et al., 2016 [1] and VanEpps & Roberto, 2016 [2]) | How much **added** **sugar** do you think is in **sweetened pre-packaged teas** like the ones above?  **[show image of teas - sweetened Lipton bottled tea, Snapple peach tea]** | 1 (None)  2  3  4  5 (A lot) |
| H115  know_unswttea  Knowledge of added sugar (non-SSBs)  (Adapted from  Roberto et al., 2016 [1] and VanEpps & Roberto, 2016 [2]) | How much **added** **sugar** do you think is in **unsweetened pre-packaged teas** like the ones above?  **[show image of teas - diet Lipton bottled tea, diet Snapple peach tea]** | 1 (None)  2  3  4  5 (A lot) |
| H120  know_flvmlk  Knowledge of added sugar (SSBs)  (Adapted from  Roberto et al., 2016 [1] and VanEpps & Roberto, 2016 [2]) | How much **added** **sugar** do you think is in **flavored milks (chocolate, strawberry)** like the ones above?  **[show image of flavored milk]** | 1 (None)  2  3  4  5 (A lot) |
| H130  know_unflvmlk  Knowledge of added sugar (non-SSBs)  (Adapted from  Roberto et al., 2016 [1] and VanEpps & Roberto, 2016 [2]) | How much **added sugar** do you think is in **plain milks** like the ones above?  **[show image of unflavored milk]** | 1 (None)  2  3  4  5 (A lot) |
|  | **F: Risk perceptions - child** |  |
| F10  chld_intl | You just bought a drink for your [child].  The next questions will ask about this child. | prompt |
| T40  ethn_chld  Hispanic ethnicity  (2010 Census [3]) | Is your [child] of Hispanic, Latino, or Spanish origin? | 1=No, not of Hispanic, Latino, or Spanish origin  2=Yes, Mexican, Mexican American, Chicano  3=Yes, Puerto Rican  4=Yes, Cuban  5=Yes, another Hispanic, Latino, or Spanish origin. Write, for example, Salvadoran, Dominican, Colombian, Guatemalan, Spaniard, Ecuadorian, etc.: _____ |
| T50  race_chld  (2010 Census [3]) | What race is your [child]? (Check all that apply) | 1= White  2= Black or African American  3= American Indian or Alaska Native  4= Asian  5= Native Hawaiian or Other Pacific Islander  6= Some other race (please specify): ______ |
| F20  prompt_9 | The next questions are about **beverages with added sugar (non-diet).** Examples of beverages with added sugar include regular sodas, fruit-flavored (not 100% juice) drinks, and pre-sweetened coffees and teas. Other examples are regular sports drinks, flavored waters, and energy drinks.  Say how much you agree with each statement below. |  |
| F30  prompt_10 | Drinking beverages with added sugar every day would increase my [child]’s risk of…  **[format otcm_hrtdam, otcm_t2d, otcm_hlth as matrix]** |  |
| F40  otcm_hrtdam  Perceived likelihood / Outcome expectations for child**  (Adapted from  Roberto et al., 2016 [1] and VanEpps & Roberto, 2016 [2]) | Heart damage | 1 = Not at all  …  5 = A lot |
| F50  otcm_t2d  Perceived likelihood / Outcome expectations for child**  (Adapted from  Roberto et al., 2016 [1] and VanEpps & Roberto, 2016 [2]) | Type 2 diabetes. | 1 = Not at all  …  5 = A lot |
| F60  otcm_hlth  Perceived likelihood / Outcome expectations for child**  (Adapted from  Roberto et al., 2016 [1] and VanEpps & Roberto, 2016 [2]) | Having health problems | 1 = Not at all  …  5 = A lot |
|  | **G: Consumption attitudes – child – Control** |  |
| G10  prompt_6 | **The next questions are also about different types of beverages.**  **[display logic, display this block IF trial_arm=barcode]**  **[page break]** |  |
| G20  prompt_7 | How **unhealthy** or **healthy** is it for your child to drink **XXX**, like the ones above?  How **unappealing** or **appealing** would your [child] find XXX, like the ones above?  How **not** **tasty** or **tasty** would your [child] find XXX, like the ones above?  [repeat prompt for each beverage] |  |
| G30  hlth_soda  SSB consumption attitudes – healthfulness**  (Adapted from Bollard et al., 2016 [4]) | **Regular (non-diet) soda or soft drinks**  **[Show image of regular sodas based on condition]** | 1= Unhealthy  …  5 = Healthy |
| G80  appl_soda  SSB attitudes – Appeal of SSBs for child**  (Adapted from Bollard et al., 2016 [4]) | **[place below Unhealthy...Healthy Q]** | 1 = Unappealing  …  5 = Appealing |
| G110  tst_soda  SSB attitudes – tastiness of SSBs for child**  (Adapted from Bollard et al., 2016 [4]) | **[place below Unappealing…Appealing Q]** | 1 = Not tasty  …  5 = Tasty |
| G40  hlth_sprt  SSB consumption attitudes – healthfulness**  (Adapted from Bollard et al., 2016 [4]) | **Regular (non-diet) sports drinks**  **[show image of regular Gatorade based on condition]** | 1= Unhealthy  …  5 = Healthy |
| G70  appl_sprt  SSB attitudes – Appeal of SSBs for child**  (Adapted from Bollard et al., 2016 [4]) | **[place below Unhealthy...Healthy Q]** | 1 = Unappealing  …  5 = Appealing |
| G120  tst_sprt  SSB attitudes – tastiness of SSBs for child**  (Adapted from Bollard et al., 2016 [4]) | **[place below Unappealing…Appealing Q]** | 1 = Not tasty  …  5 = Tasty |
| G50  hlth_flvwtr  SSB consumption attitudes – healthfulness**  (Adapted from Bollard et al., 2016 [4]) | **Regular (non-diet) flavored waters**  **[show image of regular Vitamin Water based on condition]** | 1= Unhealthy  …  5= Healthy |
| G80  appl_flvwtr  SSB attitudes – Appeal of SSBs for child**  (Adapted from Bollard et al., 2016 [4]) | **[place below Unhealthy...Healthy Q]** | 1 = Unappealing  …  5= Appealing |
| G130  tst_flvwtr  SSB attitudes – tastiness of SSBs for child**  (Adapted from Bollard et al., 2016 [4]) | **[place below Unappealing…Appealing Q]** | 1 = Not tasty  …  5= Tasty |
| G60  hlth_frtflv  SSB consumption attitudes – healthfulness**  (Adapted from Bollard et al., 2016 [4]) | **Fruit-flavored drinks** **(not 100% juice)**  **[Show image of fruit drinks based on condition]** | 1= Unhealthy  …  5 = Healthy |
| G90  appl_frtflv  SSB attitudes – Appeal of SSBs for child**  (Adapted from Bollard et al., 2016 [4]) | **[place below Unhealthy...Healthy Q]** | 1 = Unappealing  …  5 = Appealing |
| G140  tstl_frtflv  SSB attitudes – tastiness of SSBs for child**  (Adapted from Bollard et al., 2016 [4]) | **[place below Unappealing…Appealing Q]** | 1 = Not tasty  …  5 = Tasty |
| G61  hlth_swtea  SSB consumption attitudes – healthfulness**  (Adapted from Bollard et al., 2016 [4]) | **Sweetened pre-packaged teas**  **[Show image of teas based on condition]** | 1= Unhealthy  …  5 = Healthy |
| G90  appl_swtea  SSB attitudes – Appeal of SSBs for child**  (Adapted from Bollard et al., 2016 [4]) | **[place below Unhealthy...Healthy Q]** | 1 = Unappealing  …  5 = Appealing |
| G140  tst_swtea  SSB attitudes – tastiness of SSBs for child**  (Adapted from Bollard et al., 2016 [4]) | **[place below Unappealing…Appealing Q]** | 1 = Not tasty  …  5 = Tasty |
| G62  hlth_flvmlk  SSB consumption attitudes – healthfulness**  (Adapted from Bollard et al., 2016 [4]) | **Flavored milks (chocolate, strawberry)**  **[Show image of flavored milks based on condition]** | 1= Unhealthy  …  5= Healthy |
| G91  appl_flvmlk  SSB consumption attitudes – healthfulness**  (Adapted from Bollard et al., 2016 [4]) | **[place below Unhealthy...Healthy Q]** | 1 = Unappealing  …  5 = Appealing |
| G141  tst_flvmlk  SSB consumption attitudes – healthfulness**  (Adapted from Bollard et al., 2016 [4]) | **[place below Unappealing…Appealing Q]** | 1 = Not tasty  …  5 = Tasty |
|  | **G: Consumption attitudes – child – Warning Labels** |  |
| G10  prompt_6 | **The next questions are also about different types of beverages.**  **[display logic, display this block IF trial_arm=warning]**  **[page break]** |  |
| G20  prompt_7 | How **unhealthy** or **healthy** is it for your child to drink **XXX**, like the ones above?  How **unappealing** or **appealing** would your [child] find XXX, like the ones above?  How **not** **tasty** or **tasty** would your [child] find XXX, like the ones above?  [repeat prompt for each beverage] |  |
| G30  hlth_soda  SSB consumption attitudes – healthfulness**  (Adapted from Bollard et al., 2016 [4]) | **Regular (non-diet) soda or soft drinks**  **[Show image of regular sodas based on condition]** | 1= Unhealthy  …  5 = Healthy |
| G80  appl_soda  SSB attitudes – Appeal of SSBs for child**  (Adapted from Bollard et al., 2016 [4]) | **[place below Unhealthy...Healthy Q]** | 1 = Unappealing  …  5= Appealing |
| G110  tst_soda  SSB attitudes – tastiness of SSBs for child**  (Adapted from Bollard et al., 2016 [4]) | **[place below Unappealing…Appealing Q]** | 1 = Not tasty  …  5 = Tasty |
| G40  hlth_sprt  SSB consumption attitudes – healthfulness**  (Adapted from Bollard et al., 2016 [4]) | **Regular (non-diet) sports drinks**  **[show image of regular Gatorade based on condition]** | 1= Unhealthy  …  5 = Healthy |
| G70  appl_sprt  SSB attitudes – Appeal of SSBs for child**  (Adapted from Bollard et al., 2016 [4]) | **[place below Unhealthy...Healthy Q]** | 1 = Unappealing  …  5= Appealing |
| G120  tst_sprt  SSB attitudes – tastiness of SSBs for child**  (Adapted from Bollard et al., 2016 [4]) | **[place below Unappealing…Appealing Q]** | 1 = Not tasty  …  5 = Tasty |
| G50  hlth_flvwtr  SSB consumption attitudes – healthfulness**  (Adapted from Bollard et al., 2016 [4]) | **Regular (non-diet) flavored waters**  **[show image of regular Vitamin Water based on condition]** | 1= Unhealthy  …  5 = Healthy |
| G80  appl_flvwtr  SSB attitudes – Appeal of SSBs for child**  (Adapted from Bollard et al., 2016 [4]) | **[place below Unhealthy...Healthy Q]** | 1 = Unappealing  …  5= Appealing |
| G130  tst_flvwtr  SSB attitudes – tastiness of SSBs for child**  (Adapted from Bollard et al., 2016 [4]) | **[place below Unappealing…Appealing Q]** | 1 = Not tasty  …  5 = Tasty |
| G60  hlth_frtflv  SSB consumption attitudes – healthfulness**  (Adapted from Bollard et al., 2016 [4]) | **Fruit-flavored drinks (not 100% juice)**  **[Show image of fruit drinks based on condition]** | 1= Unhealthy  …  5 = Healthy |
| G90  appl_frtflv  SSB attitudes – Appeal of SSBs for child**  (Adapted from Bollard et al., 2016 [4]) | **[place below Unhealthy...Healthy Q]** | 1 = Unappealing  …  5= Appealing |
| G140  tstl_frtflv  SSB attitudes – tastiness of SSBs for child**  (Adapted from Bollard et al., 2016 [4]) | **[place below Unappealing…Appealing Q]** | 1 = Not tasty  …  5 = Tasty |
| G61  hlth_swtea  SSB consumption attitudes – healthfulness**  (Adapted from Bollard et al., 2016 [4]) | **Sweetened pre-packaged teas**  **[Show image of teas based on condition]** | 1= Unhealthy  …  5 = Healthy |
| G90  appl_swtea  SSB attitudes – Appeal of SSBs for child**  (Adapted from Bollard et al., 2016 [4]) | **[place below Unhealthy...Healthy Q]** | 1 = Unappealing  …  5= Appealing |
| G140  tst_swtea  SSB attitudes – tastiness of SSBs for child**  (Adapted from Bollard et al., 2016 [4]) | **[place below Unappealing…Appealing Q]** | 1 = Not tasty  …  5 = Tasty |
| G62  hlth_flvmlk  SSB consumption attitudes – healthfulness**  (Adapted from Bollard et al., 2016 [4]) | **Flavored milks (chocolate, strawberry)**  **[Show image of flavored milks based on condition]** | 1= Unhealthy  …  5 = Healthy |
| G91  appl_flvmlk  SSB consumption attitudes – healthfulness**  (Adapted from Bollard et al., 2016 [4]) | **[place below Unhealthy...Healthy Q]** | 1 = Unappealing  …  5= Appealing |
| G141  tst_flvmlk  SSB consumption attitudes – healthfulness**  (Adapted from Bollard et al., 2016 [4]) | **[place below Unappealing…Appealing Q]** | 1 = Not tasty  …  5 = Tasty |
|  | **N: Intentions to Limit Child’s SSB Consumption – Control** |  |
| N70  prompt_13 | **The next questions are about the next week (7 days).**  **[display logic, display this block IF trial_arm=barcode]** |  |
| N80  intent_soda  Intentions to limit SSB consumption – specific beverages**  (Adapted from Klein et al., 2009 [5]) | In the next week, how often do you plan to give your [child] **regular (non-diet) sodas or soft drinks** like the ones above?  **[Show image of sodas]** | 0= Never  1= 1 time per week  2.5= 2-3 times per week  5= 4-6 times per week  7= 1 time per day  14= 2 times per day  21= 3+ times per day |
| N90  intent_sprt  Intentions to limit SSB consumption – specific beverages**  (Adapted from Klein et al., 2009 [5]) | In the next week, how often do you plan to give your [child] **regular (non-diet) sports drinks** like the ones above?  **[show image of regular Gatorade]** | 0= Never  1= 1 time per week  2.5= 2-3 times per week  5= 4-6 times per week  7= 1 time per day  14= 2 times per day  21= 3+ times per day |
| N100  intent_flvwtr  Intentions to limit SSB consumption – specific beverages**  (Adapted from Klein et al., 2009 [5]) | In the next week, how often do you plan to give your [child] **regular (non-diet) flavored waters** like the ones above?  **[show image of regular Vitamin Water]** | 0= Never  1= 1 time per week  2.5= 2-3 times per week  5= 4-6 times per week  7= 1 time per day  14= 2 times per day  21= 3+ times per day |
| N110  intent_frtflv  Intentions to limit SSB consumption – specific beverages**  (Adapted from Klein et al., 2009 [5]) | In the next week, how often do you plan to give your [child] **fruit-flavored drinks (not 100% juice)** like the ones above?  **[show image of fruit drinks]** | 0= Never  1= 1 time per week  2.5= 2-3 times per week  5= 4-6 times per week  7= 1 time per day  14= 2 times per day  21= 3+ times per day |
| N115  intent_swttea  Intentions to limit SSB consumption – specific beverages**  (Adapted from Klein et al., 2009 [5]) | In the next week, how often do you plan to give your [child] **sweetened packaged teas** like the ones above?  **[show image of teas]** | 0= Never  1= 1 time per week  2.5= 2-3 times per week  5= 4-6 times per week  7= 1 time per day  14= 2 times per day  21= 3+ times per day |
| N120  intent_ flvmlk  Intentions to limit SSB consumption – specific beverages**  (Adapted from Klein et al., 2009 [5]) | In the next week, how often do you plan to give your [child] **flavored milks (chocolate, strawberry)** like the ones above?  **[show image of flavored milks]** | 0= Never  1= 1 time per week  2.5= 2-3 times per week  5= 4-6 times per week  7= 1 time per day  14= 2 times per day  21= 3+ times per day |
|  | **N: Intentions to Limit Child’s SSB Consumption – Warning Labels** |  |
| N70  prompt_13 | **The next statements are about the next week (7 days).**  **[page break]**  **[display logic, display this block IF trial_arm=warning]** |  |
| N80  intent_soda  Intentions to limit SSB consumption – specific beverages**  (Adapted from Klein et al., 2009 [5]) | In the next week, how often do you plan to give your [child] **regular (non-diet) sodas or soft drinks** like the ones above?  **[Show image of sodas]** | 0= Never  1= 1 time per week  2.5= 2-3 times per week  5= 4-6 times per week  7= 1 time per day  14= 2 times per day  21= 3+ times per day |
| N90  intent_sprt  Intentions to limit SSB consumption – specific beverages**  (Adapted from Klein et al., 2009 [5]) | In the next week, how often do you plan to give your [child] **regular (non-diet) sports** **drinks** like the ones above?  **[show image of regular Gatorade]** | 0= Never  1= 1 time per week  2.5= 2-3 times per week  5= 4-6 times per week  7= 1 time per day  14= 2 times per day  21= 3+ times per day |
| N100  intent_flvwtr  Intentions to limit SSB consumption – specific beverages**  (Adapted from Klein et al., 2009 [5]) | In the next week, how often do you plan to give your [child] **regular (non-diet) flavored** **waters** like the ones above?  **[show image of regular Vitamin Water]** | 0= Never  1= 1 time per week  2.5= 2-3 times per week  5= 4-6 times per week  7= 1 time per day  14= 2 times per day  21= 3+ times per day |
| N110  intent_frtflv  Intentions to limit SSB consumption – specific beverages**  (Adapted from Klein et al., 2009 [5]) | In the next week, how often do you plan to give your [child] **fruit-flavored drinks** **(not 100% juice)** like the ones above?  **[show image of fruit drinks]** | 0= Never  1= 1 time per week  2.5= 2-3 times per week  5= 4-6 times per week  7= 1 time per day  14= 2 times per day  21= 3+ times per day |
| N115  intent_swttea  Intentions to limit SSB consumption – specific beverages**  (Adapted from Klein et al., 2009 [5]) | In the next week, how often do you plan to give your [child] **sweetened packaged teas** like the ones above?  **[show image of teas]** | 0= Never  1= 1 time per week  2.5= 2-3 times per week  5= 4-6 times per week  7= 1 time per day  14= 2 times per day  21= 3+ times per day |
| N120  intent_ flvmlk  Intentions to limit SSB consumption – specific beverages**  (Adapted from Klein et al., 2009 [5]) | In the next week, how often do you plan to give your [child] **flavored milk (chocolate, strawberry)** like the ones above?  **[show image of flavored milks]** | 0= Never  1= 1 time per week  2.5= 2-3 times per week  5= 4-6 times per week  7= 1 time per day  14= 2 times per day  21= 3+ times per day |
|  | **M: Injunctive norms - child** |  |
| M10  prompt_11 | **Say how much you disagree or agree with the next statements.**  **[Format as matrix]** |  |
| M20  nrms_cog  Injunctive norms about limiting child’s SSBs**  (Adapted from Zoellner et al., 2012 [6]) | People who are important to me think my [child] should drink fewer beverages with added sugar each week. | 1 = Strongly disagree  2 = Somewhat disagree  3 = Neither agree nor disagree  4 = Somewhat agree  5 = Strongly agree |
| M30  nrms_apprv  Injunctive norms about limiting child’s SSBs**  (Adapted from Zoellner et al., 2012 [6]) | People who are important to me would approve of my [child] drinking fewer beverages with added sugar each week. | 1 = Strongly disagree  2 = Somewhat disagree  3 = Neither agree nor disagree  4 = Somewhat agree  5 = Strongly agree |
| M40  nrms_wnt  Injunctive norms about limiting child’s SSBs**  (Adapted from Zoellner et al., 2012 [6]) | People who are important to me want my [child] to drink fewer beverages with added sugar each week.  [page break] | 1 = Strongly disagree  2 = Somewhat disagree  3 = Neither agree nor disagree  4 = Somewhat agree  5 = Strongly agree |
| General belief in climate change  (Adapted from Benjamin et al., 2016 [7]) | **Say how much you agree or disagree with the following statement:**  Climate change is occurring now. | 1=Strongly disagree  2=Somewhat disagree  3=Neither agree nor disagree  4=Somewhat agree  5=Strongly agree |
|  | **C: Message reactions – Experimental labels** |  |
| C05  Programming Note | **[display label on top of all pages with questions about the label]** |  |
| C30  prompt_2 | **Above are pictures of labels (stickers) that were on some of the beverages in the store. The next questions are about these labels.**  **[Insert image warning labels for experimental group]** |  |
|  | **[attention question separate]**  **[matrix and randomize emotions]**  **[elab in matrix]**  **[PME as a matrix]**  **[avoidance separate]**  **[learn something separate]**  **[control separate]** |  |
| C50  att_lab  Attention to label**  (Adapted from Davis et al., 2011 [8]) | How much do these labels grab your attention? | 1=Not at all  2=Very little  3=Somewhat  4=Quite a bit  5=A great deal |
| C70  neg_anx  Negative emotions**  (Adapted from Davis et al., 2011 [8]) | How much do these labels make you feel **anxious**? | 1=Not at all  2=Very little  3=Somewhat  4=Quite a bit  5=A great deal |
| C80  neg_scrd  Negative emotions**  (Adapted from Davis et al., 2011 [8]) | How much do these labels make you feel **scared**? | 1=Not at all  2=Very little  3=Somewhat  4=Quite a bit  5=A great deal |
| C90  neg_gult  Negative emotions**  (Adapted from Davis et al., 2011 [8]) | How much do these labels make you feel **guilty**? | 1=Not at all  2=Very little  3=Somewhat  4=Quite a bit  5=A great deal |
| C100  cog_lab  Thinking about warning message**  (Adapted from Fathelrahman et al., 2010 [9] and Hammond et al., 2003 [10]) | How much do these labels make you think about the health problems caused by drinking beverages with added sugar?  **[page break]** | 1=Not at all  2=Very little  3=Somewhat  4=Quite a bit  5=A great deal |
| C110  talk_lab  Anticipated social interactions** | How likely are you to talk about these labels with others in the next week?  **[page break]** | 1 = Not at all likely  2 = A little likely  3 = Somewhat likely  4 = Very likely  5 = Extremely likely |
| C120  pme_disc  Perceived message effectiveness – discouragement (list as other outcome)  (Baig et al., 2019 [11]) | How much do these labels discourage you from wanting to buy beverages with added sugar for your [child]?  **[page break]** | 1=Not at all  2=Very little  3=Somewhat  4=Quite a bit  5=A great deal |
| C121  pme_conc  Perceived message effectiveness – concerned (list as other outcome)  (Baig et al., 2019 [11]) | How much do these labels make you concerned about the health effects of your [child] drinking beverages with added sugar? | 1=Not at all  2=Very little  3=Somewhat  4=Quite a bit  5=A great deal |
| C122  pme_unpl  Perceived message effectiveness – unpleasant (list as other outcome)  (Baig et al., 2019 [11]) | How much do these labels make buying beverages with added sugar for your [child] seem unpleasant to you?  **[page break]** | 1=Not at all  2=Very little  3=Somewhat  4=Quite a bit  5=A great deal |
| C130  lab_avoid  Avoid (list as other outcome)  (Adapted from the Population Assessment of Tobacco and Health (PATH) Study [12]) | How much do you want to avoid looking at these labels?  **[page break]** | 1=Not at all  2=Very little  3=Somewhat  4=Quite a bit  5=A great deal |
| C140  lab_learn  learn  (Adapted from Pepper et al., 2020) | Did you learn something new from these labels?  [randomize order of yes and no]  **[page break]** | 1 = Yes  0 = No |
| C160  lab_cntrl  Felt more in control (%)**  (Adapted from Acton & Hammond, 2018 [13]) | Overall, would these labels make you feel…  **[page break]** | 1=Less in control of making healthy eating decisions  2=Neither less nor more in control of making healthy eating decisions  3=More in control of making healthy eating decisions |
|  | **C: Message reactions – barcode** |  |
| C05  Programming Note | **[display label on top of all pages with questions about the label]**  **[only show these questions for the control group]** |  |
| C20  ntcng_c  Noticing – Control arm** | Some of the beverages in the store may have had extra white rectangle labels (stickers) added on top of the regular packaging, like in this picture. Did you see these labels?  **[Show picture of generic soda bottle with blank label located where we placed labels in the store]**  **[page break]** | 0 = No  1 = Yes |
| C30  prompt_2 | **Above is a picture of a label (sticker) that was on some of the beverages in the store. The next questions are about this label.** |  |
|  | **[randomize order of C50-C100; matrix]** |  |
| C50  att_lab  Attention to label**  (Adapted from Davis et al., 2011 [8]) | How much does this label grab your attention? | 1=Not at all  2=Very little  3=Somewhat  4=Quite a bit  5=A great deal |
| C70  neg_anx  Negative emotions**  (Adapted from Davis et al., 2011 [8]) | How much does this label make you feel **anxious**? | 1=Not at all  2=Very little  3=Somewhat  4=Quite a bit  5=A great deal |
| C80  neg_scrd  Negative emotions**  (Adapted from Davis et al., 2011 [8]) | How much does this label make you feel **scared**? | 1=Not at all  2=Very little  3=Somewhat  4=Quite a bit  5=A great deal |
| C90  neg_gult  Negative emotions**  (Adapted from Davis et al., 2011 [8]) | How much does this label make you feel **guilty**? | 1=Not at all  2=Very little  3=Somewhat  4=Quite a bit  5=A great deal |
| C100  cog_lab  Thinking about warning message**  (Adapted from Fathelrahman et al., 2010 [9] and Hammond et al., 2003 [10]) | How much does this label make you think about the health problems caused by drinking beverages with added sugar?  **[page break]** | 1=Not at all  2=Very little  3=Somewhat  4=Quite a bit  5=A great deal |
| C110  talk_lab  Anticipated social interactions** | How likely are you to talk about this label with others in the next week?  **[page break]** | 1 = Not at all likely  2 = A little likely  3 = Somewhat likely  4 = Very likely  5 = Extremely likely |
| C120  pme_disc  Perceived message effectiveness – discouragement (list as other outcome)  (Baig et al., 2019 [11]) | How much does this label discourage you from wanting to buy beverages with added sugar for your [child]?  **[page break]** | 1=Not at all  2=Very little  3=Somewhat  4=Quite a bit  5=A great deal |
| C121  pme_conc  Perceived message effectiveness – concerned (list as other outcome)  (Baig et al., 2019 [11]) | How much does this label make you concerned about the health effects of your [child] drinking beverages with added sugar?  **[page break]** | 1=Not at all  2=Very little  3=Somewhat  4=Quite a bit  5=A great deal |
| C122  pme_unpl  Perceived message effectiveness – unpleasant (list as other outcome)  (Baig et al., 2019 [11])) | How much does this label make buying beverages with added sugar for your [child] seem unpleasant to you?  **[page break]** | 1=Not at all  2=Very little  3=Somewhat  4=Quite a bit  5=A great deal |
| C130  lab_avoid  Avoid (list as other outcome)  (Adapted from the Population Assessment of Tobacco and Health (PATH) Study [12]) | How much do you want to avoid looking at this label?  **[page break]** | 1=Not at all  2=Very little  3=Somewhat  4=Quite a bit  5=A great deal |
| C140  lab_learn  learn  (Adapted from Pepper et al., 2020 [14]) | Did you learn something new from this label?  **[randomize order of yes and no]** | 1 = Yes  0 = No |
| C160  lab_cntrl  Felt more in control (%)**  (Adapted from Acton & Hammond, 2018 [13]) | Overall, would this label make you feel…  **[page break]** | 1=Less in control of making healthy eating decisions  2=Neither less nor more in control of making healthy eating decisions  3=More in control of making healthy eating decisions |
|  | **P: Policy** |  |
| C170  prompt_15 | **The next question is about health warning labels. Lawmakers in five states in the U.S. have proposed requiring health warning labels on beverages with added sugar. Below are examples of health warning labels.**  **[insert warning labels – even for control group]** |  |
| C180  policy_spprt  (Hall et al., 2018 [15]) | Would you oppose or support a law requiring health warnings like these to appear on beverages with added sugar? | 1 = Strongly oppose  2 = Somewhat oppose  3 = Somewhat support  4 = Strongly support |
|  | **T: Demographics** |  |
| T10  prompt_16 | **The next questions are about you.**  **[page break]** |  |
| T20  lang_gen  English proficiency (SASH [16]) | In general, what language(s) do you read and speak? | 1 = Only English  2 = English better than Spanish  3 = Both equally  4 = Spanish better than English  5 = Only Spanish |
| T30  lang_home  English proficiency (SASH [16]) | What language do you usually speak at home? | 1 = Only English  2 = More English than Spanish  3 = Both equally  4 = More Spanish than English  5 = Only Spanish |
| T40  lang_think  English proficiency (SASH [16]) | In what language do you usually think? | 1 = Only English  2 = More English than Spanish  3 = Both equally  4 = More Spanish than English  5 = Only Spanish |
| T50  lang_frnd  English proficiency (SASH [16]) | What language do you usually speak with your friends? | 1 = Only English  2 = More English than Spanish  3 = Both equally  4 = More Spanish than English  5 = Only Spanish |
| T120  gndr  gender  (Williams Institute [17]) | How do you describe your gender identity? | 1= Man  2= Woman  3= Other gender identity (please specify): _____ |
| T130  age | How old are you? Enter your age in years. | [numeric free response, restricted to 18-99] |
| T140  ethnct_hisp  Hispanic ethnicity  (2010 Census [3]) | Are you of Hispanic, Latino, or Spanish origin? | 1=No, not of Hispanic, Latino, or Spanish origin  2=Yes, Mexican, Mexican American, Chicano  3=Yes, Puerto Rican  4=Yes, Cuban  5=Yes, another Hispanic, Latino, or Spanish origin. Write, for example, Salvadoran, Dominican, Colombian, Guatemalan, Spaniard, Ecuadorian, etc.: _____ |
| T150  race  (2010 Census [3]) | What is your race? (Check all that apply) | 1= White  2= Black or African American  3= American Indian or Alaska Native  4= Asian  5= Native Hawaiian or Other Pacific Islander  6= Some other race (please specify): ______ |
| T160  sexorient  Sexual orientation  (NHANES [18]) | Do you think of yourself as… | 1= Heterosexual or straight (that is, sexually attracted only to [men/women; program based on T120]  2= Homosexual or gay (that is, sexually attracted only to [men/women; program based on response to T120]  3= Bisexual (that is, sexually attracted to men and women)  4= Something else |
| T170  met_pref  Metric preference | When you think about your height and weight, do you prefer to use… | 1=feet and pounds  2=meters and kilograms |
| T180  height_f  Height, feet | **[Ask if met_pref=1]**  How tall are you? Please enter your height in **feet** and **inches** using the menus below.  **[Drop down menu for feet with the following options: 3, 4, 5, 6, 7, 8. Label this drop -down menu as “Feet”]** | 3 = 3 feet  4 = 4 feet  5 = 5 feet  6 = 6 feet  7 = 7 feet |
| T190  height_i  Height, inches | **[Show on same page as above – separate drop-down menu for inches with the following options: 0, 1, 2, 3, 4, 5, 6, 7, 8, 9, 10, 11. Label this drop-down menu as “Inches”]** | 1 = 1 inch  2 = 2 inches  3 = 3 inches  4 = 4 inches  5 = 5 inches  6 = 6 inches  7 = 7 inches  8 = 8 inches  9 = 9 inches  10 = 10 inches  11 = 11 inches |
| T200  height_m  Height, meters | **[Ask if met_pref=2]**  How tall are you? Enter your height in **meters**. | [Numerical free response. Allow entries > 1.0m and <2.3m]. |
| T210  weight_lb  Weight, lbs | **[Ask if met_pref=1]**  How much do you weigh? Enter your weight in **pounds**. | [Numerical free response. Allow entries > 40lbs and <900lbs]. |
| T215  weight_kg  Weight, kg | **[Ask if met_pref=2]**  How much do you weigh? Enter your weight in **kilograms**. | [Numerical free response. Allow entries > 18kg and <408kg]. |
| T216  NFP prompt | **The next question will ask about information on the back of food and drink packages.** |  |
| T220  nfp_use  NFP | **[display NFP and ingredient list from a fruit drink]**  How often do you use this type of information when deciding to buy a food or beverage? | 1= Never  2= Rarely  3= Sometimes  4= Often  5= All the time |
| T225  nfp_liter  Literacy | How often do you need to have someone help you when you read instructions, pamphlets, or other written material from your doctor or pharmacy? | 1=Never 2=Sometimes 3=Often 4=Always |
| Education  educ | What is the highest level of school you have completed? | 1=Less than high school or U.S. high school equivalent (GED)  2=High school diploma or U.S. high school equivalent (GED)  3=4-year college degree  4= Master’s degree, graduate degree, or more |
| T240  income_hsh  Poverty  (Adapted from the Population Assessment of Tobacco and Health (PATH) Study [12]) | Which of the following categories best describes your total household income in the last 12 months? | 1= Less than $10,000  2= $10,000 to $14,999  3= $15,000 to $24,999  4= $25,000 to $34,999  5= $35,000 to $49,999  6= $50,000 to $74,999  7= $75,000 to $99,999  8= $100,000 to $149,999  9= $150,000 to $199,999  10= $200,000 or more |
| T250  num_hsh  Number in household  (USDHHS 2016 [19]) | How many people depend on this income, including you? | # of people [restricted to 1-20] |

**References**

1. Roberto CA, Wong D, Musicus A, Hammond D. The Influence of Sugar-Sweetened Beverage Health Warning Labels on Parents' Choices. Pediatrics. 2016;137(2):e20153185. Epub 2016/01/16. doi: 10.1542/peds.2015-3185. PubMed PMID: 26768346.

2. VanEpps EM, Roberto CA. The Influence of Sugar-Sweetened Beverage Warnings: A Randomized Trial of Adolescents' Choices and Beliefs. Am J Prev Med. 2016;51(5):664-72. Epub 2016/09/13. doi: 10.1016/j.amepre.2016.07.010. PubMed PMID: 27617366; PubMed Central PMCID: PMCPMC5533079.

3. Questions Planned for the 2010 Census and American Community Survey: U.S. Department of Commerce, Economics and Statistics Administration, U.S. Census Bureau; 2008 [cited 2020]. Available from: <https://www2.census.gov/programs-surveys/acs/operations_admin/Questions_Planned_for_the_2010_Census_and_American_Community_Survey.pdf>.

4. Bollard T, Maubach N, Walker N, Ni Mhurchu C. Effects of plain packaging, warning labels, and taxes on young people's predicted sugar-sweetened beverage preferences: an experimental study. Int J Behav Nutr Phys Act. 2016;13(1):95. Epub 2016/09/02. doi: 10.1186/s12966-016-0421-7. PubMed PMID: 27580589; PubMed Central PMCID: PMCPMC5007687.

5. Klein WM, Zajac LE, Monin MM. Worry as a moderator of the association between risk perceptions and quitting intentions in young adult and adult smokers. Ann Behav Med. 2009;38(3):256-61. Epub 2010/01/06. doi: 10.1007/s12160-009-9143-2. PubMed PMID: 20049660.

6. Zoellner J, Estabrooks PA, Davy BM, Chen YC, You W. Exploring the theory of planned behavior to explain sugar-sweetened beverage consumption. J Nutr Educ Behav. 2012;44(2):172-7. Epub 2011/12/14. doi: 10.1016/j.jneb.2011.06.010. PubMed PMID: 22154130; PubMed Central PMCID: PMCPMC3290682.

7. Benjamin D, Por H-H, Budescu D. Climate Change Versus Global Warming: Who Is Susceptible to the Framing of Climate Change? Environment and Behavior. 2016;49(7):745-70. doi: 10.1177/0013916516664382.

8. Davis KC, Nonnemaker JM, Farrelly MC, Niederdeppe J. Exploring differences in smokers' perceptions of the effectiveness of cessation media messages. Tob Control. 2011;20(1):26-33. Epub 2010/09/21. doi: 10.1136/tc.2009.035568. PubMed PMID: 20852323.

9. Fathelrahman AI, Omar M, Awang R, Cummings KM, Borland R, Bin Mohd Samin AS. Impact of the new Malaysian cigarette pack warnings on smokers' awareness of health risks and interest in quitting smoking. Int J Environ Res Public Health. 2010;7(11):4089-99. Epub 2010/12/09. doi: 10.3390/ijerph7114089. PubMed PMID: 21139879; PubMed Central PMCID: PMCPMC2996227.

10. Hammond D, Fong GT, McDonald PW, Cameron R, Brown KS. Impact of the graphic Canadian warning labels on adult smoking behaviour. Tob Control. 2003;12(4):391-5. Epub 2003/12/09. doi: 10.1136/tc.12.4.391. PubMed PMID: 14660774; PubMed Central PMCID: PMCPMC1747800.

11. Baig SA, Noar SM, Gottfredson NC, Boynton MH, Ribisl KM, Brewer NT. UNC Perceived Message Effectiveness: Validation of a Brief Scale. Ann Behav Med. 2019;53(8):732-42. Epub 2018/10/16. doi: 10.1093/abm/kay080. PubMed PMID: 30321252; PubMed Central PMCID: PMCPMC6636889.

12. Population Assessment of Tobacco and Health Study. PATH: Population Assessment of Tobacco and Health 2014 [cited 2014 April 3]. Available from: <http://www.pathstudyinfo.nih.gov/UI/HomeMobile.aspx>.

13. Acton RB, Hammond D. Do Consumers Think Front-of-Package "High in" Warnings are Harsh or Reduce their Control? A Test of Food Industry Concerns. Obesity (Silver Spring). 2018;26(11):1687-91. Epub 2018/10/04. doi: 10.1002/oby.22311. PubMed PMID: 30281206.

14. Pepper JK, Nguyen Zarndt A, Eggers ME, Nonnemaker JM, Portnoy DB. Influence of Warning Statements on Understanding of the Negative Health Consequences of Smoking. Nicotine Tob Res. 2020;22(10):1805-15. Epub 2020/03/24. doi: 10.1093/ntr/ntaa031. PubMed PMID: 32202620.

15. Hall MG, Marteau TM, Sunstein CR, Ribisl KM, Noar SM, Orlan EN, et al. Public support for pictorial warnings on cigarette packs: an experimental study of US smokers. J Behav Med. 2018;41(3):398-405. Epub 2018/02/08. doi: 10.1007/s10865-018-9910-2. PubMed PMID: 29411272; PubMed Central PMCID: PMCPMC5924634.

16. Marin G, Sabogal F, Marin BV, Otero-Sabogal R, Perez-Stable EJ. Development of a Short Acculturation Scale for Hispanics. Hispanic Journal of Behavioral Sciences. 1987;9(2):183-205. doi: 10.1177/07399863870092005.

17. Badgett MVL, Baker KE, Conron KJ, Gates GJ, Gill A, Greytak E, et al. Best Practices for Asking Questions to Identify Transgender and Other Gender Minority Respondents on Population-Based Surveys (GenIUSS): UCLA School of Law Williams Institute; 2014. Available from: <https://williamsinstitute.law.ucla.edu/publications/geniuss-trans-pop-based-survey/>.

18. Questionnaires, Datasets, and Related Documentation: Centers for Disease Control and Prevention, National Center for Health Statistics, National Health and Nutrition Examination Survey; [cited 2019]. Available from: <https://wwwn.cdc.gov/nchs/nhanes/>.

19. 2016 Poverty Guidelines. Washington, D.C.: Office of the Assistant Secretary for Planning and Evaluation, U.S. Department of Health and Human Services; 2016. Available from: <https://aspe.hhs.gov/2016-poverty-guidelines>.
