# Supplementary material for: The impact of pictorial health warnings on purchases of sugary drinks for children: A randomized controlled trial
Source: PLoS Med. 2022 Feb 1;19(2):e1003885. doi: 10.1371/journal.pmed.1003885 (PMC8806063; doi:10.1371/journal.pmed.1003885)
Supplement: S3 Table — Interaction p-values from logistic regression models. No p-values were statistically significant after applying Holm–Bonferroni correction. *p from Wald test for joint interaction. (DOCX) [file pmed.1003885.s004.docx]

**S3 Table**. **Percent of parents selecting sugary drinks by trial arm. Interaction *p*-values from logistic regression models.** No *p*-values were statistically significant after applying Holm-Bonferroni correction. **p* from Wald test for joint interaction.

| **Participant characteristics** | **Control** | **Pictorial** | **Difference** | ***p* for interaction** |
| --- | --- | --- | --- | --- |
| Age |  |  |  | 0.507 |
| 18-39 years old | 46% | 26% | -20% |  |
| 40 years old + | 44% | 31% | -13% |  |
| Gender |  |  |  | 0.033 |
| Man | 54% | 16% | -38% |  |
| Woman | 43% | 32% | -11% |  |
| Sexual orientation |  |  |  | 0.599 |
| Straight or heterosexual | 46% | 29% | -17% |  |
| Gay, lesbian, bisexual, another  orientation | 50% | 21% | -29% |  |
| Race/ethnicity |  |  |  | 0.045* |
| Non-Hispanic white | 42% | 26% | -16% |  |
| Non-Hispanic non-white | 56% | 34% | -22% |  |
| Hispanic | 29% | 24% | -6% |  |
| Educational attainment |  |  |  | 0.863 |
| Less than four year college degree | 47% | 30% | -17% |  |
| Four year college degree or more | 45% | 27% | -18% |  |
| Annual household income |  |  |  | 0.916 |
| $0-$49,999 | 45% | 29% | -17% |  |
| $50,000 or more | 45% | 27% | -18% |  |
| Nutrition Facts Panel use |  |  |  | 0.718 |
| Never, rarely, sometimes | 51% | 35% | -16% |  |
| Often, all the time | 39% | 22% | -17% |  |
| Frequency of needing help reading  medical information |  |  |  | 0.852 |
| Never | 45% | 29% | -17% |  |
| Sometimes, often, always | 40% | 27% | -13% |  |
| Language of survey administration |  |  |  | 0.389 |
| English | 46% | 28% | -19% |  |
| Spanish | 35% | 30% | -5% |  |
| Age of child the parent shopped for, in years |  |  |  | 0.559 |
| 2-5 | 46% | 25% | -21% |  |
| 6-12 | 45% | 30% | -15% |  |
| Gender of child the parent shopped for |  |  |  | 0.630 |
| Boy | 47% | 32% | -15% |  |
| Girl | 44% | 25% | -19% |  |
| Child’s consumption of sugary drinks |  |  |  | 0.760 |
| Below sample median | 35% | 20% | -15% |  |
| At or above sample median | 52% | 37% | -15% |  |
| Time of participation |  |  |  | 0.537 |
| Pre-pandemic | 44% | 31% | -13% |  |
| During pandemic | 46% | 27% | -19% |  |
