# Supplementary material for: The impact of pictorial health warnings on purchases of sugary drinks for children: A randomized controlled trial
Source: PLoS Med. 2022 Feb 1;19(2):e1003885. doi: 10.1371/journal.pmed.1003885 (PMC8806063; doi:10.1371/journal.pmed.1003885)
Supplement: S1 Study Protocol — (PDF) [file pmed.1003885.s005.pdf]

Impact of Warnings on Sugar-Sweetened Beverages

Study Protocol and Informed Consent

NCT #04223687

Version Date: 6/14/2021

PROTOCOL TITLE: Protocol for a study examining the impact of warnings on sugar-sweetened beverages

Lead Investigator:

Marissa G. Hall, PhD

University of North Carolina at Chapel Hill

I confirm that I have read this protocol and understand it.

Principal Investigator Name: Marissa G. Hall

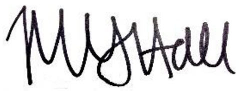

Principal Investigator Signature: ____________________

Date: June 14th, 2021

TABLE OF CONTENTS

Table of Contents 3
Protocol Synopsis 4
BACKGROUND AND RATIONALE 6
STUDY OBJECTIVE 7
INVESTIGATIONAL PLAN (BRIEF OVERVIEW) 7
STUDY PROCEDURES (WHAT WILL BE DONE) 8
SCREENING AND MONITORING EVALUATIONS AND MEASUREMENTS (HOW MEASUREMENTS WILL BE MADE) 9
STATISTICAL CONSIDERATION 14
SAFETY MANAGEMENT 16
DATA COLLECTION AND MANAGMENT 16
RECRUITMENT STRATEGY 16
CONSENT PROCESS 16
PLANS FOR PUBLICATION 17
REFERENCES 17
INFORMED CONSENT FORMS 19
JANUARY 2020-MARCH 2020 19
NOVEMBER 2020-FEBRUARY 2021 22
FEBRUARY 2021-MARCH 2021 25

PROTOCOL SYNOPSIS

**Study Title**	Randomized control trial evaluating the impact of warnings on sugar-sweetened beverages
**Funder**	Robert Wood Johnson Foundation<br>NIH National Heart, Lung, and Blood Institute
**Study Rationale**	High intake of added sugars is a major contributor to diet-related chronic diseases including obesity, heart disease, type II diabetes, and cancer. A potential policy to lower consumption of added sugar is the addition of pictorial warning labels to sugar-sweetened beverages (SSBs). This study examined the impact of pictorial health warning labels on parents' product selection for one of their children ages 2-12 years old.
Study Objective(s)	Primary<br>• To examine the impact of pictorial health warning labels on parents' decisions to purchase an SSB in a randomized controlled trial in a naturalistic convenience store lab.<br>Secondary<br>• To determine the impact of pictorial warning labels on perceptions about and reactions to SSBs.
**Study Design**	Participants will provide written (pre-COVID) or verbal (post-COVID) informed consent to participate in the study. Study staff will randomly assign participants to one of the two study arms, and randomization will be determined a priori. The participants will visit the naturalistic convenience store lab and be given a shopping task to select 1 beverage and 1 snack to purchase for their child ages 2-12 (if more than one child 2-12, the study staff will randomly select one of the children), as well as 1 household good. After completing the shopping task, the participant will complete a survey in Qualtrics. The survey will ask a series of questions about the beverage and warning labels (e.g., perceived healthfulness, perceived appeal, perceived amount of added sugar in SSB). Questions will also include standard demographic and health related variables.
**Subject Population**	Inclusion Criteria<br>1. 18 years old or older

key criteria for Inclusion and Exclusion:	2. Be able to read and speak English or Spanish<br>3. Be able to take a survey on a computer or tablet in English or Spanish<br>4. Be the parent or guardian to at least one child between the ages of 2 and 12 who consumed at least one SSB in the week prior to taking the screener<br>Exclusion Criteria<br>1. Cannot have participated in the investigator's previous study at the naturalistic convenience store lab<br>2. Cannot live in the same household as another enrolled study participant
Number Of Subjects	326
Study Duration	Each subject's participation will last approximately 45-60 minutes<br>Prior to COVID-19, the entire study was expected to last 6 months. Due to pandemic-related delays, the entire study is expected to last 15 months.
Study Phases<br>Screening<br>RCT	(1) Screening: screening for eligibility and obtaining consent.<br>(2) Randomization: Randomly assigning participants to one of two conditions.<br>(3) Shopping task: Instruct participants to complete a shopping task in a naturalistic convenience store lab.<br>(4) Survey: Direct participants to a Qualtrics survey measuring perceptions and reactions as well as standard demographics.
Statistical And Analytic Plan	We will descriptively report unadjusted means and percentages for the primary and secondary outcomes. We will run chi-squared tests (for dichotomous variables) and independent sample t-tests (for continuous variables) to test our hypotheses. We will report results when controlling for any participant demographic characteristics found to be unbalanced across treatment arms in balance tests, if these results differ substantively from unadjusted results. We will conduct exploratory analysis to test whether a variety of characteristics moderate the effect of SSB health warnings on SSB purchase likelihood and will fit a series of logistic regression models, with trial arm, the moderator, and their interaction as predictors.
Data And Safety Monitoring Plan	Participants will be assigned an identification number, and all data will be de-identified. Researchers will store the data linking the participant and the identification number on a remote terminal server, and only research investigators and staff will have access to the data. All hardcopy research files will be kept in locked files in a locked room in a locked building.

BACKGROUND AND RATIONALE

Introduction

Childhood obesity is a major public health problem in the US among racial and ethnic minorities, including among the US's growing Latino population. Latino children are at much higher risk of obesity than white children; 26% of Latino children ages 2-19 are obese compared to 14% of white children.[1] Sugar-sweetened beverage (SSB) consumption is a major driver of the childhood obesity epidemic.[2-5] Daily consumption of SSBs is higher among Latino children ages 2-19 (67%) than white children (58%).[6] Policies, systems, and environmental strategies are urgently needed to reduce SSB consumption and obesity among Latino children. One promising but understudied policy for addressing childhood obesity is requiring health warnings on the front of SSB containers. This study will assess the impact of pictorial health warnings in an RCT in a naturalistic convenience store lab (UNC Mini Mart).

Health warnings (i.e., direct statements about the health harms associated with consuming a product) on cigarette packs reduce smoking[7] and are required in over 150 countries.[8] SSB health warnings in the US are a viable childhood obesity prevention policy option at the local, state, and federal level.[9,10] Five US states have proposed laws requiring health warnings on SSBs, and Congress has regulatory authority to pass legislation requiring a federal agency to implement SSB health warnings, as they did for cigarette warnings.[11] The US Food and Drug Administration has demonstrated an interest in stronger SSB labels, commissioning two reviews of front-of-package nutrition labels.[12,13] However, almost no research has focused on health warnings, instead studying other types of labels such as numeric labels or nutrient warnings.[14] Gathering evidence on SSB health warnings will ensure that future policies are evidence-based and therefore likely to withstand legal challenges.

Pictorial warnings are especially promising for reducing childhood obesity among Latinos. Language barriers in the Latino community, especially among the 17.5 million Latinos in the US with limited English proficiency,[15] may limit the impact of text-only warnings. Pictorial health warnings, which include text statements as well as photographs or icons, may be able to overcome education and language disparities by conveying health harms with words and pictures.[16,17] However, most previous studies on pictorial health warnings have ignored minority populations, and studies focused on design elements have not considered how to develop pictorial warnings that are meaningful to diverse populations including Latinos.

The impact of pictorial health warnings in real-world food retail environments on SSB purchasing behavior is unknown. Most research on health warnings to date has focused on self-reported outcomes. For example, our team has conducted online experiments that suggest that SSB warnings – particularly those that include images, describe health effects, and use stronger causal language – are perceived as more effective.[18,19] Other studies have shown that SSB warnings decrease hypothetical intentions to purchase SSBs.[20-22] Although this evidence is suggestive, few studies have examined SSB warnings in real-world food retail environments. Evidence on whether pictorial health warnings reduce actual purchases of SSBs is critical for understanding whether these warnings will help prevent obesity.

Our proposed project aims to design and evaluate pictorial health warnings on SSB purchases, with a long-term goal of informing policies that can improve diet, prevent obesity, and ultimately prevent type 2 diabetes and other cardiometabolic diseases among Latino children.

Randomized controlled trials (RCTs) are needed to test the causal impact of nutrient warnings on decisions to purchase an SSB high in added sugar. The project will address a major gap by evaluating the impact of pictorial health warnings on SSB purchasing behavior in a real-world retail environment.

Potential Risks and Benefits

There are no direct benefits for participating this research study. The potential risks to participating in this study include (1) discomfort while answering the surveys, (2) loss of privacy resulting from accidental disclosure of responses to survey questions or of data collected on purchases, and (3) exposure to COVID-19. For information on how the study team mitigated these risks, please see the Safety Management section (page 13).

STUDY OBJECTIVE

The purpose of this study is to determine whether pictorial warnings decrease parents' SSB purchases for their children in a real-world RCT.

Primary Outcome

Percentage of participants who purchase an SSB for their child.

Secondary Outcomes

- Total calories purchased from SSBs
- Intention to give SSBs to child
- Percentage of participants who noticed trial label
- Thinking about harms of drinking SSBs
- Negative affective reactions
- Anticipated social interactions
- Percentage of participants who felt more in control of healthy eating decisions
- Perceived amount of added sugar in SSBs
- Perceived likelihood of child experiencing health problems due to sugar sweetened beverages
- Healthfulness of SSBs for child
- Appeal of SSBs for child
- Tastiness of SSBs for child
- Injunctive norms about limiting child's SSBs

Other Outcomes

- Anticipated avoidance of label
- Perceived message effectiveness
- Policy support

INVESTIGATIONAL PLAN (brief overview)

Study Design

The study design is a between-subjects randomized controlled trial. Study staff will randomly assign participants to one of the two study arms where they will see one of two labels on the SSB upon entering the naturalistic convenience store lab (UNC Mini Mart). Randomization order will be

determined a priori, and participants will have an equal chance of being randomized to either arm of the trial.

Study Arms:

1. Control (neutral barcode label)
2. Health warning labels: one warning label with an image of a necrotic foot along with the text "WARNING: Excess consumption of drinks with added sugar contributes to type 2 diabetes," and another warning label with an image of a damaged heart with the text "WARNING: Excess consumption of drinks with added sugar contributes to heart damage."

In the UNC Mini Mart, participants will be asked to complete a shopping task for their child aged 2-12 (if more than one child aged 2-12, the study staff will randomly select one child for the participant to shop for). They will select one beverage and one snack for their child, along with one household good. The SSBs in the UNC Mini Mart will all have control or health warning labels affixed to the packaging, depending on the study arm. Participants will be informed that the checkout counter will randomly select one of the three products to take home. In reality, participants will always take home the beverage they select. After completing the shopping tasks, participants will complete a Qualtrics survey on a computer or tablet measuring reactions to and perceptions of the SSBs and labels as well as standard demographics. Upon completion, participants will go home with their selected beverage and a cash incentive of $38 (if beverage cost $2) or $39 (if beverage cost $1).

Study Duration, Enrollment and Number of Subjects

Participation in the study will last approximately 45-60 minutes. The entire study was initially expected to last 6 months but was postponed during data collection due to the COVID-19 pandemic. Due to this, the entire study is expected to last approximately 15 months.

Study Population

Study population is non-Hispanic (50-80%) and Hispanic (20-50%) parents of children between the ages of 2-12 who consume sugar sweetened beverages.

Inclusion Criteria

1. 18 years old or older
2. Able to read and speak English or Spanish
3. Able to take a survey on a computer or tablet in English or Spanish
4. Parent or guardian of at least one child between the ages of 2 and 12 who consumed at least one SSB in the week prior to taking the screener

Exclusion Criteria

1. Participated in the investigators' previous study at the naturalistic convenience store lab
2. Lives in the same household as another enrolled study participant

STUDY PROCEDURES (what will be done)

Study Steps

Study staff will recruit potential participants through a variety of methods, including in-person (pre-COVID-19), social media, Craigslist, and email distribution lists. If interested, potential participants will complete a screener in Qualtrics to see if they are eligible. If they are eligible, study staff will contact the potential participants to confirm that they are still interested in participating. If the participant is still interested, study staff will schedule an appointment.

After scheduling an appointment, the study staff reviews the consent form with the participant. Before the COVID-19 pandemic, participants would provide their informed consent after arrival to the study visit. To reduce COVID-19 risk, study staff will review the consent form with the participant over the phone at the time of scheduling and the participant will provide verbal consent.

Prior to the appointment, study staff will set up the store in the correct randomly assigned experimental condition by displaying the SSBs with the appropriate labels. When the participant arrives, the study staff will consent the participant (pre-COVID-19) or conduct a COVID-19 screening. The study staff will then instruct the participant to shop for one beverage and one snack for their child who is between 2-12 years old, as well as one household good. If they have more than one child aged 2-12, the study staff will inform the participant which child to shop for, which is determined by randomly selecting one of the children at the time of scheduling. After selecting these three items, the study staff will inform the participant that the computer will randomly select one item for the participant to take home. However, all participants will take home the beverage. After completion of the shopping task, the participant will take a Qualtrics survey on a tablet or computer. In the Qualtrics survey, participants will answer questions about their perceptions about and reactions to the SSBs and labels, as well as standard demographic questions (see measurements section for more detail). Upon completing the survey, participants will receive their incentive ($38 or $39 + beverage, for a total value of $40) and a handout about SSBs and healthy beverage choices for their child(ren).

Subject Completion/ Withdrawal procedures

A study participant is determined to have completed the study when they have finished the shopping task and completed the study survey. To withdraw their data from the study, a participant would contact the study team or the university IRB.

Screen failure procedures

Prior to entering into the study, individuals will complete a survey screener. If the individual is younger than 18 years old, does not have a child aged 2-12 who consumed at least one SSB in the week prior to taking the screener, is not able to read and speak English or Spanish, or is not able to complete a survey on a tablet or computer in English or Spanish, they will be deemed ineligible. Such participants will not be contacted by study staff. If they reach out to the study team, they will be informed that they are not eligible to participate.

SCREENING AND MONITORING EVALUATIONS AND MEASUREMENTS (how measurements will be made)

Measurements will include results from the selection task as well as subjective responses by participants. For the primary outcomes, study staff will record the beverage selected by participants.

Secondary outcomes will include a variety of measurements including participant responses to 5-point and 7-point Likert scale questions and yes/no questions.

Question	Response Scale
**Secondary outcomes**	
In the next week, how often do you plan to give your [child] **regular (non-diet) sodas or soft drinks** like the ones above?<br><br>**[Show image of sodas]**<br><br>REPEAT QUESTION FOR:<br>• **regular (non-diet) sports drinks**<br>• **regular (non-diet) flavored waters**<br>• **fruit-flavored drinks (not 100% juice)**<br>• **sweetened packaged teas**<br>• **flavored milks (chocolate, strawberry)**	0=Never<br>1=1 time per week<br>2.5=2-3 times per week<br>5=4-6 times per week<br>7=1 time per day<br>14=2 times per day<br>21=3+ times per day
**Control Arm:**<br><br>**[Show picture of generic soda bottle with barcode label located where we placed labels in the store]**<br><br>Some of the beverages in the store may have had extra white rectangle labels (stickers) added on top of the regular packaging, like in these pictures above. Did you see these labels?<br><br>**Warning Arm:**<br><br>**[Show picture of generic soda bottle with warning labels located where we placed labels in the store]**<br><br>Some of the beverages in the store may have had extra picture warning labels (stickers) on top of the regular packaging, like in these pictures above. Did you see these labels?	0=No<br>1=Yes
How much do these labels make you think about the health problems caused by drinking beverages with added sugar?	1=Not at all<br>2=Very little<br>3=Somewhat<br>4=Quite a bit<br>5=A great deal
How much do these labels make you feel **anxious**?	1=Not at all<br>2=Very little<br>3=Somewhat<br>4=Quite a bit<br>5=A great deal
How much do these labels make you feel **scared**?	1=Not at all<br>2=Very little<br>3=Somewhat<br>4=Quite a bit

	5=A great deal
How much do these labels make you feel **guilty**?	1=Not at all<br>2=Very little<br>3=Somewhat<br>4=Quite a bit<br>5=A great deal
How likely are you to talk about these labels with others in the next week?	1=Not at all likely<br>2=A little likely<br>3=Somewhat likely<br>4=Very likely<br>5=Extremely likely
Overall, would these labels make you feel…	1=<u>Less</u> in control of making healthy eating decisions<br>2=<u>Neither</u> less nor more in control of making healthy eating decisions<br>3=<u>More</u> in control of making healthy eating decisions
How much **added sugar** do you think is in **regular (non-diet) soda and soft drinks** like the ones above?<br><br>[show image of regular sodas with warning label or barcode, based on trial arm]	1=None<br>2<br>3<br>4<br>5=A lot

REPEAT QUESTION FOR:

- **Diet soda and soft drinks**
- **Regular (non-diet) sports drinks**
- **Diet sports drinks**
- **Regular (non-diet) flavored waters**
- **Diet flavored waters**
- **Bottled waters**
- **Fruit-flavored drinks (not 100% juice)**
- **100% fruit juice**
- **Sweetened pre-packaged teas**
- **Unsweetened pre-packaged teas**
- **Flavored milks (chocolate, strawberry)**
- **Plain milks**

Drinking beverages with added sugar every day would increase <u>my [child]'s</u> risk of having health problems.	1=Not at all<br>2<br>3<br>4<br>5=A lot
How **unhealthy** or **healthy** is it for <u>your child</u> to drink [beverage], like the ones above?	1=Unhealthy<br>2<br>3<br>4<br>5=Healthy

Ask question for the following beverages:

- **Regular (non-diet) soda or soft drinks**
- **Regular (non-diet) sports drinks**
- **Regular (non-diet) flavored waters**
- **Fruit-flavored drinks (not 100% juice)**
- **Sweetened pre-packaged teas**
- **Flavored milks (chocolate, strawberry)**

How **unappealing** or **appealing** is it for your child to drink [beverage], like the ones above?

1=Unappealing
2
3
4
5=Appealing

Ask question for the following beverages:

- **Regular (non-diet) soda or soft drinks**
- **Regular (non-diet) sports drinks**
- **Regular (non-diet) flavored waters**
- **Fruit-flavored drinks (not 100% juice)**
- **Sweetened pre-packaged teas**

Flavored milks (chocolate, strawberry)

How **not tasty** or **tasty** is it for your child to drink [beverage], like the ones above?

1=Not tasty
2
3
4
5=Tasty

Ask question for the following beverages:

- **Regular (non-diet) soda or soft drinks**
- **Regular (non-diet) sports drinks**
- **Regular (non-diet) flavored waters**
- **Fruit-flavored drinks (not 100% juice)**
- **Sweetened pre-packaged teas**
- **Flavored milks (chocolate, strawberry)**

Say how much you disagree or agree with the next statements.

People who are important to me think my [child] should drink fewer beverages with added sugar each week.

1=Strongly disagree
2=Somewhat disagree
3=Neither agree nor disagree
4=Somewhat agree
5=Strongly agree

People who are important to me would approve of my [child] drinking fewer beverages with added sugar each week.

1=Strongly disagree
2=Somewhat disagree
3=Neither agree nor disagree
4=Somewhat agree
5=Strongly agree

People who are important to me want my [child] to drink fewer beverages with added sugar each week.	1=Strongly disagree<br>2=Somewhat disagree<br>3=Neither agree nor disagree<br>4=Somewhat agree<br>5=Strongly agree
**Other outcomes**	
How much do you want to avoid looking at these labels?	1=Not at all<br>2=Very little<br>3=Somewhat<br>4=Quite a bit<br>5=A great deal
How much do these labels discourage you from wanting to buy beverages with added sugar for your [child]?	1=Not at all<br>2=Very little<br>3=Somewhat<br>4=Quite a bit<br>5=A great deal
How much do these labels make you concerned about the health effects of your [child] drinking beverages with added sugar?	1=Not at all<br>2=Very little<br>3=Somewhat<br>4=Quite a bit<br>5=A great deal
How much do these labels make buying beverages with added sugar for your [child] seem unpleasant to you?	1=Not at all<br>2=Very little<br>3=Somewhat<br>4=Quite a bit<br>5=A great deal
**The next question is about health warning labels. Lawmakers in five states in the U.S. have proposed requiring health warning labels on beverages with added sugar. Below are examples of health warning labels.**<br>**[insert intervention arm warnings – even for control group]**<br>Would you oppose or support a law requiring health warnings like these to appear on beverages with added sugar?	1=Strongly oppose<br>2=Somewhat oppose<br>3=Somewhat support<br>4=Strongly support

STATISTICAL CONSIDERATION

Statistical Methods

Main analyses

We will use a two-sided critical alpha of 0.05 to conduct all statistical tests. All confidence intervals presented will be 95% and two-sided. Analyses of the primary and secondary outcomes will include all randomized participants according to the trial arm they were randomized to receive (i.e., intent-to-treat). We will use complete case analysis to handle any missing data.

To prepare the data, we will first examine all scales to ensure adequate internal consistency (i.e., Cronbach's alpha > 0.70), dropping items as needed to improve consistency. If we are unable to achieve adequate internal consistency by dropping items, we may exclude the unreliable scales from analyses (e.g., not analyze treatment effects on these outcomes).

We will descriptively report unadjusted means and percentages for the primary and secondary outcomes. For significance testing of our hypotheses, we will run chi-squared tests for dichotomous variables and independent samples t-tests for continuous variables.

We will also report results when controlling for any participant demographic characteristics found to be unbalanced across treatment arms in balance tests, if these results differ substantively from unadjusted results (i.e., changes in statistical significance or direction of effect). These analyses will use linear regression for continuous outcomes, logistic regression for dichotomous outcomes, two-part models for zero-inflated outcomes (e.g., SSB calories) and a count model for the number of SSBs purchased.

<u>**Exploratory analyses of the primary outcome**</u>

We will examine whether the following participant characteristics moderate the effect of SSB health warnings on likelihood of purchasing SSBs:

a) Age category of parent (in years);
b) Age category of child (2-5 vs. 6-12);
c) Gender of parent (man vs. woman);
d) Gender of child (boy vs. girl);
e) Sexual orientation (gay, lesbian, or bisexual vs. not);
f) Race of parent (white vs. non-white);
g) Ethnicity of parent (Hispanic/Latino vs. non-Hispanic/Latino)
h) Low educational attainment (some college or less vs. college or more),
i) Nutrition Facts Panel use (will dichotomize to create roughly equivalent groups based on frequencies);
j) Frequency of needing help reading medical information (will dichotomize to create roughly equivalent groups based on frequencies);
k) Household income ($50,000 or more vs. less than $50,000);
l) Child's frequency of consuming SSBs (above vs. at or below the sample median);
m) Language participant took the survey in (Spanish vs. English)

To test whether these characteristics moderate the effect of SSB health warnings on SSB purchase likelihood, we will fit a series of logistic regressions models (one for each potential moderator), with trial arm, the moderator, and their interaction as predictors. We will probe significant interactions by calculating the marginal effect of health warnings on the outcome at different levels of the moderating variable. Moderation analyses will use a Bonferroni-corrected *p*-value.

Sample Size and Power

The primary objective of this trial is to evaluate the effect of SSB health warnings on parents' likelihood of purchasing an SSB for their child. We used G*Power3 to determine sample size needs for addressing this objective. We based this off of the most similar study to date in terms of methods and stimuli,[23] which found an effect size of d=.32 for the effect of warnings vs. control on likelihood of purchasing

SSBs. Using these specifications and a two-sided alpha of 0.05, we determined a necessary sample of 314 to detect an effect of d=.32 or larger with 80% power. To account for potential missing data or incomplete study visits, we aim to enroll 326 participants (163 in each arm).

SAFETY MANAGEMENT

There is risk of exposure to COVID-19 for participants who enroll after March 2020. Several precautions will be taken to protect against this risk. To minimize in-person contact, the informed consent process will take place over the phone prior to the study visit. One day before the study visit and upon arrival, all participants will be screened for COVID-19, and the visit will be rescheduled if the participant has any symptoms. The study staff will maintain at least a 6-to-10-foot distance from the participant at all times. The participant will be required to wear a mask and gloves, and the study staff will be required to wear a mask, gloves, and face shield. There will only be one team member working at a time, and there will be only one participant at the UNC Mini Mart at a time. All surfaces will be cleaned between visits, and the UNC Mini Mart will be equipped with a HEPA air filter. Study staff will screen themselves for COVID-19 symptoms before coming to work, and they will not come to work if they do not pass the screening. The study team consulted with the UNC IRB, Office of Occupational Health and Safety, and departmental leadership to develop COVID-19 safety protocols.

DATA COLLECTION AND MANAGMENT

To protect identifying information that will be collected, data for all participants will be kept strictly confidential. All research files will be kept in locked files in a locked room in a locked building. Participants will be assigned an identification number, and all data will be de-identified. Names and other identifiers will be maintained separately and only study staff members will have access to the linked file. Study data will be placed on a secure remote serve that is not linked to the internet, and only study personnel will be granted remote access to this secure server.

RECRUITMENT STRATEGY

To recruit for this study, we will send emails to relevant listservs and post advertisements on Craigslist and social media. We will also post flyers in community venues and reach out to partner organizations (e.g., El Centro Hispano) to find eligible participants. Prior to the COVID-19 pandemic, we will also conduct in-person recruitment at community venues, such as churches, community centers, bus stops and restaurants.

CONSENT PROCESS

To provide consent, individuals will first complete a survey screener in Qualtrics. If they are eligible for the study, study staff will contact the participant to schedule an appointment. Prior to the COVID-19 pandemic, we will complete the informed consent at the beginning of the appointment. Study staff will review the consent form with the participant, and the participant will sign the form to indicate their consent to participate. After the COVID-19 pandemic, we will complete the informed consent over the phone at the time of scheduling. The study staff will email the form to the participant, and they will review the form together. The participant will verbally inform the study staff of their consent to participate.

PLANS FOR PUBLICATION

The investigators plan to submit this study as a peer-reviewed paper. They will target public health peer-reviewed journals for manuscript submission.

REFERENCES

1. Hales CM, Carroll MD, Fryar CD, Ogden CL. Prevalence of obesity among adults and youth: United States, 2015-2016. In. Hyattsville, MD: National Center for Health Statistics; 2017.
2. Hu FB. Resolved: there is sufficient scientific evidence that decreasing sugar-sweetened beverage consumption will reduce the prevalence of obesity and obesity-related diseases. *Obes Rev.* 2013;14(8):606-619.
3. Malik VS, Popkin BM, Bray GA, Despres JP, Hu FB. Sugar-sweetened beverages, obesity, type 2 diabetes mellitus, and cardiovascular disease risk. *Circulation.* 2010;121(11):1356-1364.
4. Malik VS, Schulze MB, Hu FB. Intake of sugar-sweetened beverages and weight gain: a systematic review. *Am J Clin Nutr.* 2006;84(2):274-288.
5. Te Morenga L, Mallard S, Mann J. Dietary sugars and body weight: systematic review and meta-analyses of randomised controlled trials and cohort studies. *British Medical Journal.* 2013;346:e7492
6. Bleich SN, Vercammen KA, Koma JW, Li Z. Trends in Beverage Consumption Among Children and Adults, 2003-2014. *Obesity (Silver Spring).* 2018;26(2):432-441.
7. Brewer NT, Hall MG, Noar SM, et al. Effect of pictorial cigarette pack warnings on changes in smoking behavior: A randomized clinical trial. *JAMA Intern Med.* 2016;176(7):905-912.
8. *Cigarette package health warnings: International status report*. Ontario, Canada: Canadian Cancer Society; 2016.
9. Jacobson MF, Krieger J, Brownell KD. Potential policy approaches to address diet-related diseases. *JAMA.* 2018.
10. Construction of Pub. L. 101–535, § 6(c); 104 Stat. 2364 (Nov. 8, 1990); codified 21 U.S.C. Sec. 343-1. In.
11. United States Public Laws. *Family smoking prevention and tobacco control act of 2009. 111th Congress, 1st Session. Public Law 111-31 [H.R. 1256].* 2009.
12. Institute of Medicine. *Examination of front-of-package nutrition rating systems and symbols: Phase 1 report.* Washington, DC: National Academies of Science;2010.
13. Institute of Medicine. *Front-of-package nutrition rating systems and symbols: Promoting healthier choices.* Washington, DC: National Academies of Science;2011.
14. Cecchini M, Warin L. Impact of food labelling systems on food choices and eating behaviours: a systematic review and meta-analysis of randomized studies. *Obes Rev.* 2016;17(3):201-210.
15. Flores A. 2015, Hispanic Population in the United States Statistical Portrait. Pew Research Center. https://www.pewresearch.org/hispanic/2017/09/18/2015-statistical-information-on-hispanics-in-united-states/#hispanic-language. Published 2017. Accessed July 23, 2018.
16. Hammond D, Fong GT, Borland R, Cummings KM, McNeill A, Driezen P. Communicating risk to smokers: the impact of health warnings on cigarette packages. *American Journal of Preventive Medicine.* 2007;32(3):202.
17. Thrasher JF, Villalobos V, Szklo A, et al. Assessing the impact of cigarette package health warning labels: a cross-country comparison in Brazil, Uruguay and Mexico. *Salud publica de Mexico.* 2010;52 Suppl 2:S206-215.
18. Grummon AH, Hall MG, Taillie LS, Brewer NT. How should sugar-sweetened beverage health warnings be designed? A randomized experiment. *Preventive Medicine.* Under Review.

19. Hall MG, Grummon AH, Maynard OM, Kameny MR, Jenson D, Popkin BM. Investigating the strength of causal language used in health warning labels: A study of US adults. *Journal of Behavioral Medicine.* Under Review.
20. VanEpps EM, Roberto CA. The influence of sugar-sweetened beverage warnings: A randomized trial of adolescents' choices and beliefs. *Am J Prev Med.* 2016;51(5):664-672.
21. Bollard T, Maubach N, Walker N, Ni Mhurchu C. Effects of plain packaging, warning labels, and taxes on young people's predicted sugar-sweetened beverage preferences: an experimental study. *The international journal of behavioral nutrition and physical activity.* 2016;13(1):95.
22. Roberto CA, Wong D, Musicus A, Hammond D. The influence of sugar-sweetened beverage health warning labels on parents' choices. *Pediatrics.* 2016;137(2):e20153185.
23. Grummon AH, Smith NR, Golden SD, Frerichs L, Taillie LS, Brewer NT. Health Warnings on Sugar-Sweetened Beverages: Simulation of Impacts on Diet and Obesity Among U.S. Adults. *Am J Prev Med.* 2019;57(6):765-774.

Informed Consent Forms

January 2020-March 2020

University of North Carolina at Chapel Hill
Consent to Participate in a Research Study
Adult Participants for Randomized Controlled Trial

Consent Form Version Date: March 3, 2020
IRB Study # 19-0277
Title of Study: Evaluating consumer responses to policies
Principal Investigator: Marissa Hall
Principal Investigator Department: Health Behavior
Principal Investigator Phone number: (919) 407-8590
Principal Investigator Email Address: storestudy@unc.edu
Co-Principal Investigator: Lindsey Smith Taillie
Co-Principal Investigator Contact Information: (919) 407-8590
Funding Source and/or Sponsor: Robert Wood Johnson Foundation Healthy Eating Research Group

Concise Summary. We would like to invite you to take part in a research study to better understand the factors that affect consumers' purchasing decisions in a convenience store environment. This study is open to parents and caregivers of children ages 2-12.
You will participate in this study for the duration of one visit which will last approximately 45 to 60 minutes. You will complete a shopping task in the UNC Mini Mart, where you will have to select one drink and one snack for your child, as well as one household good. We will randomly select one of the items for you to take home. The total cost of the item you take home will be subtracted from your cash incentive. After you complete the shopping task, you will take a computer survey. The remainder of your $40 incentive will be given to you in the form of cash.
Research is designed to benefit society by gaining new knowledge. You will not benefit personally from being in this research study. The two potential risks to participating in this study include (1) discomfort while answering the surveys and (2) loss of privacy resulting from accidental disclosure of responses to survey questions or of data collected on purchases. However, we have taken steps to limit the probability of these risks from occurring.
If you are interested in learning more about this study, please continue reading below.

<u>What are some general things you should know about research studies?</u>

You are being asked to take part in a voluntary research study. You may choose not to participate, or you may withdraw your consent to be in the study, for any reason, without penalty.

Research studies are designed to obtain new knowledge. This new information may help people in the future. You may not receive any direct benefit from being in the research study. There also may be risks to being in research studies.

Details about this study are discussed below. It is important that you understand this information so that you can make an informed choice about being in this research study.

You will be given a copy of this consent form. You should ask the researchers named above, or staff members who may assist them, any questions you have about this study at any time.

<u>What is the purpose of this study?</u>

The purpose of this research study is to understand the factors that affect consumers' purchasing decisions in a

convenience store environment. You are being asked to be in the study because you are a parent or caregiver to at least one child age 2-12.

How many people will take part in this study?

A total of approximately 350 people will participate in this research study.

How long will your part in this study last?

Your participation in this study will consist of one visit to the UNC Mini Mart in Chapel Hill, NC. Your visit will last approximately 45 to 60 minutes.

In the rare case that your survey data do not save, we will contact you by phone to collect your demographic information, such as questions about your education level, income, and gender.

What will happen if you take part in the study?

- Randomization: We will assign you to a study group. The study groups will see differences in some aspects of the store. You will not know which group you are in. Both groups will take a survey after the shopping task is complete.
- Shopping task: You will be asked to complete a shopping task in the UNC Mini Mart (1700 MLK Jr. Blvd, Chapel Hill, NC, 27514). You will be required to select one beverage and one snack for one of your children aged 2-12 and one household good from the UNC Mini Mart. One of the items will be randomly selected for you to take home.
- Survey: After you have completed the shopping task, you will be asked to complete a survey on a computer. You may choose to skip questions in the survey for any reason.

What are the possible benefits from being in this study?

Research is designed to benefit society by gaining new knowledge. You will not benefit personally from being in this research study.

What are the possible risks or discomforts involved from being in this study?

The two potential risks to participating in this study include (1) discomfort while answering the surveys and (2) loss of privacy resulting from accidental disclosure of responses to survey questions or of data collected on purchases. There may be uncommon or previously unknown risks. You should report any problems to the researcher.

Not only are these risks small, but the consequences of a rare accidental disclosure would also be small because we are not collecting sensitive information. If these data were disclosed, they would not affect insurability, social standing, or employment. In this way, data collected by the study do not pose any risk greater than what participants encounter in their daily life.

To protect against the risk of potential loss of confidentiality:

- All research files will be kept in locked files in a locked room in a locked building.
- You will be assigned an identification number, and all data will be de-identified.
- Names and other identifiers will be maintained separately, and minimal study staff will have access to the linked file.
- Study data will be placed on a secure volume on a UNC data server that is not linked to the internet.

What if we learn about new findings or information during the study?

You will be given any new information gained during the course of the study that might affect your willingness to continue your participation.

How will information about you be protected?

Your responses to survey questions will be confidential. We will assign you a unique numerical identifier to be used on all study documents (e.g., completed surveys). We will shred any paper copies of your contact information and maintain an electronic version of your name, identifier, and phone number on a secure, password-protected server that can only be accessed by study personnel. We will delete your personal information from the computer six months after the study ends. If you indicated that you were interested in

hearing about future research studies, we will maintain your contact information indefinitely, separate from any other study data.

Participants will not be identified in any report or publication about this study. We may use de-identified data from this study in future research without additional consent.

Although every effort will be made to keep research records private, there may be times when federal or state law requires the disclosure of such records, including personal information. This is very unlikely, but if disclosure is ever required, UNC-Chapel Hill will take steps allowable by law to protect the privacy of personal information. In some cases, your information in this research study could be reviewed by representatives of the University, research sponsors, or government agencies (for example, the FDA) for purposes such as quality control or safety.

What if you want to stop before your part in the study is complete?

You can withdraw from this study at any time, without penalty. The investigators also have the right to stop your participation at any time. This could be because the entire study has been stopped. If you withdraw from the study, you will receive the standard study incentive, as described below.

Will you receive anything for being in this study?

You will receive one item (a snack, beverage, or a household good) and cash for a total value of $40. The total value of the item you receive from the UNC Mini Mart will be deducted from the starting value of $40. For example, if your item is worth $3, you would receive $37 in cash, for a total value of $40. To participate in this study, you will always take one item home from the mini mart; therefore, you will always receive less than $40 in cash. We will also provide a voucher so that you do not need to pay for parking.

Will it cost you anything to be in this study?

It will not cost you anything to be in this study. You may have to pay for transportation to the study office. You will not be reimbursed for this cost, but you will receive a parking pass if needed.

Who is sponsoring this study?

This research is funded by Healthy Eating Research, a program of the Robert Wood Johnson Foundation.

What if you have questions about this study?

You have the right to ask, and have answered, any questions you may have about this research. If you have questions about the study (including payments), complaints, concerns, or if a research-related injury occurs, you should contact the researchers listed on the first page of this form.

What if you have questions about your rights as a research participant?

All research on human volunteers is reviewed by a committee that works to protect your rights and welfare. If you have questions or concerns about your rights as a research subject, or if you would like to obtain information or offer input, you may contact the Institutional Review Board at 919-966-3113 or by email to IRB_subjects@unc.edu.

Participant's Agreement:

I have read the information provided above. I have asked all the questions I have at this time. I voluntarily agree to participate in this research study.

____ _______________

Signature of Research Participant Date

____

Printed Name of Research Participant

____ ________________

Signature of Research Team Member Obtaining Consent Date

____

Printed Name of Research Team Member Obtaining Consent

November 2020-February 2021

University of North Carolina at Chapel Hill
Consent to Participate in a Research Study
Adult Participants for Randomized Controlled Trial

Consent Form Version Date: September 25, 2020
IRB Study # 19-0277
Title of Study: Evaluating consumer responses to policies
Principal Investigator: Marissa Hall
Principal Investigator Department: Health Behavior
Principal Investigator Phone number: (919) 407-8590
Principal Investigator Email Address: storestudy@unc.edu
Co-Principal Investigator: Lindsey Smith Taillie
Co-Principal Investigator Contact Information: (919) 407-8590
Funding Source and/or Sponsor: Robert Wood Johnson Foundation Healthy Eating Research Group

Concise Summary. We would like to invite you to take part in a research study to better understand the factors that affect consumers' purchasing decisions in a convenience store environment. This study is open to parents and caregivers of children ages 2-12.

You will participate in this study for the duration of one visit which will last approximately 45 to 60 minutes. You will complete a shopping task in the UNC Mini Mart, where you will have to select one drink and one snack for your child, as well as one household good. We will randomly select one of the items for you to take home. The total cost of the item you take home will be subtracted from your cash incentive. After you complete the shopping task, you will take a computer survey. The remainder of your $40 incentive will be given to you in the form of cash.

Research is designed to benefit society by gaining new knowledge. You will not benefit personally from being in this research study. The three potential risks to participating in this study include (1) discomfort while answering the surveys, (2) loss of privacy resulting from accidental disclosure of responses to survey questions or of data collected on purchases, and (3) exposure to COVID-19. However, we have taken steps to limit the probability of these risks from occurring.

If you are interested in learning more about this study, please continue reading below.

<u>What are some general things you should know about research studies?</u>

You are being asked to take part in a voluntary research study. You may choose not to participate, or you may withdraw your consent to be in the study, for any reason, without penalty.

Research studies are designed to obtain new knowledge. This new information may help people in the future. You may not receive any direct benefit from being in the research study. There also may be risks to being in research studies.

Details about this study are discussed below. It is important that you understand this information so that you can make an informed choice about being in this research study.

You will be given a copy of this consent form. You should ask the researchers named above, or staff members who may assist them, any questions you have about this study at any time.

What is the purpose of this study?
The purpose of this research study is to understand the factors that affect consumers' purchasing decisions in a convenience store environment. You are being asked to be in the study because you are a parent or caregiver to at least one child age 2-12.

How many people will take part in this study?
A total of approximately 350 people will participate in this research study.

How long will your part in this study last?
Your participation in this study will consist of one visit to the UNC Mini Mart in Chapel Hill, NC. Your visit will last approximately 45 to 60 minutes.
In the rare case that your survey data do not save, we will contact you by phone to collect your demographic information, such as questions about your education level, income, and gender.

What will happen if you take part in the study?

- Randomization: We will assign you to a study group. The study groups will see differences in some aspects of the store. You will not know which group you are in. Both groups will take a survey after the shopping task is complete.
- Shopping task: You will be asked to complete a shopping task in the UNC Mini Mart (1700 MLK Jr. Blvd, Chapel Hill, NC, 27514). You will be required to select one beverage and one snack for one of your children aged 2-12 and one household good from the UNC Mini Mart. One of the items will be randomly selected for you to take home.
- Survey: After you have completed the shopping task, you will be asked to complete a survey on a computer. You may choose to skip questions in the survey for any reason.

What are the possible benefits from being in this study?
Research is designed to benefit society by gaining new knowledge. You will not benefit personally from being in this research study.

What are the possible risks or discomforts involved from being in this study?
The three potential risks to participating in this study include (1) discomfort while answering the surveys, (2) loss of privacy resulting from accidental disclosure of responses to survey questions or of data collected on purchases and (3) exposure to COVID-19. There may be uncommon or previously unknown risks. You should report any problems to the researcher.
The risks of discomfort and loss of privacy are small. The consequences of a rare accidental disclosure would also be small because we are not collecting sensitive information. If these data were disclosed, they would not affect insurability, social standing, or employment. In this way, data collected by the study do not pose any risk greater than what participants encounter in their daily life.
To protect against the risk of potential loss of confidentiality:

- All research files will be kept in locked files in a locked room in a locked building.
- You will be assigned an identification number, and all data will be de-identified.
- Names and other identifiers will be maintained separately, and minimal study staff will have access to the linked file.
- Study data will be placed on a secure volume on a UNC data server that is not linked to the internet.

There is risk of exposure to COVID-19. To protect against this risk:

- One day before the study visit and upon arrival, all participants will be screened for COVID-19. If you have any symptoms, we will reschedule your visit.
- The study staff will maintain a 6-foot distance from you at all times.

- You and the study staff will be required to wear a mask and gloves.
- There will only be one team member working at a time. Additionally, there will only be one participant at the UNC Mini Mart at a time.
- All surfaces will be cleaned between visits, and the UNC Mini Mart is equipped with a HEPA air filter.
- Study members will screen themselves for COVID-19 symptoms before coming to work. If they have a symptom, they will not come to work.
- If a study team member gets a COVID-19 test, they will not return to work until the test comes back negative. If they test positive for COVID-19 at any time, all participants who visited the store after the team member's potential exposure until they stopped working will be notified.

What if we learn about new findings or information during the study?

You will be given any new information gained during the course of the study that might affect your willingness to continue your participation.

How will information about you be protected?

Your responses to survey questions will be confidential. We will assign you a unique numerical identifier to be used on all study documents (e.g., completed surveys). We will shred any paper copies of your contact information and maintain an electronic version of your name, identifier, and phone number on a secure, password-protected server that can only be accessed by study personnel. We will delete your personal information from the computer six months after the study ends. If you indicated that you were interested in hearing about future research studies, we will maintain your contact information indefinitely, separate from any other study data.

Participants will not be identified in any report or publication about this study. We may use de-identified data from this study in future research without additional consent.

Although every effort will be made to keep research records private, there may be times when federal or state law requires the disclosure of such records, including personal information. This is very unlikely, but if disclosure is ever required, UNC-Chapel Hill will take steps allowable by law to protect the privacy of personal information. In some cases, your information in this research study could be reviewed by representatives of the University, research sponsors, or government agencies (for example, the FDA) for purposes such as quality control or safety.

What if you want to stop before your part in the study is complete?

You can withdraw from this study at any time, without penalty. The investigators also have the right to stop your participation at any time. This could be because the entire study has been stopped. If you withdraw from the study, you will receive the standard study incentive, as described below.

Will you receive anything for being in this study?

You will receive one item (a snack, beverage, or a household good) and cash for a total value of $40. The total value of the item you receive from the UNC Mini Mart will be deducted from the starting value of $40. For example, if your item is worth $3, you would receive $37 in cash, for a total value of $40. To participate in this study, you will always take one item home from the mini mart; therefore, you will always receive less than $40 in cash. We will also provide a voucher so that you do not need to pay for parking.

Will it cost you anything to be in this study?

It will not cost you anything to be in this study. You may have to pay for transportation to the study office. You will not be reimbursed for this cost, but you will receive a parking pass if needed.

Who is sponsoring this study?

This research is funded by Healthy Eating Research, a program of the Robert Wood Johnson Foundation.

What if you have questions about this study?

You have the right to ask, and have answered, any questions you may have about this research. If you have questions about the study (including payments), complaints, concerns, or if a research-related injury occurs, you should contact the researchers listed on the first page of this form.

What if you have questions about your rights as a research participant?
All research on human volunteers is reviewed by a committee that works to protect your rights and welfare. If you have questions or concerns about your rights as a research subject, or if you would like to obtain information or offer input, you may contact the Institutional Review Board at 919-966-3113 or by email to IRB_subjects@unc.edu.

Participant's Agreement:

I have read the information provided above. I have asked all the questions I have at this time. I give my verbal consent to participate in this research study.

Printed Name of Research Participant

____

Signature (RA) | Date

___________________________ ___________________________

February 2021-March 2021

University of North Carolina at Chapel Hill
Consent to Participate in a Research Study
Adult Participants for Randomized Controlled Trial

Consent Form Version Date: February 3, 2021
IRB Study # 19-0277
Title of Study: Evaluating consumer responses to policies
Principal Investigator: Marissa Hall
Principal Investigator Department: Health Behavior
Principal Investigator Phone number: (919) 407-8590
Principal Investigator Email Address: storestudy@unc.edu
Co-Principal Investigator: Lindsey Smith Taillie
Co-Principal Investigator Contact Information: (919) 407-8590
Funding Source and/or Sponsor: Robert Wood Johnson Foundation Healthy Eating Research Group

Concise Summary. We would like to invite you to take part in a research study to better understand the factors that affect consumers' purchasing decisions in a convenience store environment. This study is open to parents and caregivers of children ages 2-12.
You will participate in this study for the duration of one visit which will last approximately 45 to 60 minutes. You will complete a shopping task in the UNC Mini Mart, where you will have to select one drink and one snack for your child, as well as one household good. We will randomly select one of the items for you to take home. The total cost of the item you take home will be subtracted from your cash incentive. After you complete the shopping task, you will take a computer survey. The remainder of your $40 incentive will be given to you in the form of cash.

Research is designed to benefit society by gaining new knowledge. You will not benefit personally from being in this research study. The three potential risks to participating in this study include (1) discomfort while answering the surveys, (2) loss of privacy resulting from accidental disclosure of responses to survey questions or of data collected on purchases, and (3) exposure to COVID-19. However, we have taken steps to limit the probability of these risks from occurring.
If you are interested in learning more about this study, please continue reading below.

What are some general things you should know about research studies?

You are being asked to take part in a voluntary research study. You may choose not to participate, or you may withdraw your consent to be in the study, for any reason, without penalty.

Research studies are designed to obtain new knowledge. This new information may help people in the future. You may not receive any direct benefit from being in the research study. There also may be risks to being in research studies.

Details about this study are discussed below. It is important that you understand this information so that you can make an informed choice about being in this research study.

You will be given a copy of this consent form. You should ask the researchers named above, or staff members who may assist them, any questions you have about this study at any time.

What is the purpose of this study?

The purpose of this research study is to understand the factors that affect consumers' purchasing decisions in a convenience store environment. You are being asked to be in the study because you are a parent or caregiver to at least one child age 2-12.

How many people will take part in this study?

A total of approximately 350 people will participate in this research study.

How long will your part in this study last?

Your participation in this study will consist of one visit to the UNC Mini Mart in Chapel Hill, NC. Your visit will last approximately 45 to 60 minutes.
In the rare case that your survey data do not save, we will contact you by phone to collect your demographic information, such as questions about your education level, income, and gender.

What will happen if you take part in the study?

- Randomization: We will assign you to a study group. The study groups will see differences in some aspects of the store. You will not know which group you are in. Both groups will take a survey after the shopping task is complete.
- Shopping task: You will be asked to complete a shopping task in the UNC Mini Mart (1700 MLK Jr. Blvd, Chapel Hill, NC, 27514). You will be required to select one beverage and one snack for one of your children aged 2-12 and one household good from the UNC Mini Mart. One of the items will be randomly selected for you to take home.
- Survey: After you have completed the shopping task, you will be asked to complete a survey on a computer. You may choose to skip questions in the survey for any reason.

What are the possible benefits from being in this study?

Research is designed to benefit society by gaining new knowledge. You will not benefit personally from being in this research study.

What are the possible risks or discomforts involved from being in this study?

The three potential risks to participating in this study include (1) discomfort while answering the surveys, (2) loss of privacy resulting from accidental disclosure of responses to survey questions or of data collected on

purchases and (3) exposure to COVID-19. There may be uncommon or previously unknown risks. You should report any problems to the researcher.

The risks of discomfort and loss of privacy are small. The consequences of a rare accidental disclosure would also be small because we are not collecting sensitive information. If these data were disclosed, they would not affect insurability, social standing, or employment. In this way, data collected by the study do not pose any risk greater than what participants encounter in their daily life.

To protect against the risk of potential loss of confidentiality:

- All research files will be kept in locked files in a locked room in a locked building.
- You will be assigned an identification number, and all data will be de-identified.
- Names and other identifiers will be maintained separately, and minimal study staff will have access to the linked file.
- Study data will be placed on a secure volume on a UNC data server that is not linked to the internet.

There is risk of exposure to COVID-19. To protect against this risk:

- One day before the study visit, you will be screened for COVID-19. If you have any symptoms, we will reschedule your visit. We will conduct the same symptom check and a temperature check when you arrive, before you enter the building
- The study staff will maintain 10 feet or more of distance from participants whenever possible.
- You and the study staff will be required to wear a mask and gloves.
- There will only be one team member working at a time. Additionally, there will only be one participant at the UNC Mini Mart at a time.
- All surfaces will be cleaned between visits, and the UNC Mini Mart is equipped with a HEPA air filter.
- Study team members will screen themselves for COVID-19 symptoms before coming to work. If they have a symptom, they will not come to work.
- If a study member gets tested for COVID-19 because they are having symptoms, they will not return to the study site until they have a negative test result and symptoms are gone.
- If a study team member tests positive for COVID-19 at any time, or has a close contact with someone who tested positive, they will not return to the study site until they have met all quarantine requirements per guidance from UNC Environmental Health and Safety.

What if we learn about new findings or information during the study?

You will be given any new information gained during the course of the study that might affect your willingness to continue your participation.

How will information about you be protected?

Your responses to survey questions will be confidential. We will assign you a unique numerical identifier to be used on all study documents (e.g., completed surveys). We will shred any paper copies of your contact information and maintain an electronic version of your name, identifier, and phone number on a secure, password-protected server that can only be accessed by study personnel. We will delete your personal information from the computer six months after the study ends. If you indicated that you were interested in hearing about future research studies, we will maintain your contact information indefinitely, separate from any other study data.

Participants will not be identified in any report or publication about this study. We may use de-identified data from this study in future research without additional consent.

Although every effort will be made to keep research records private, there may be times when federal or state law requires the disclosure of such records, including personal information. This is very unlikely, but if disclosure is ever required, UNC-Chapel Hill will take steps allowable by law to protect the privacy of personal information. In some cases, your information in this research study could be reviewed by representatives of the University, research sponsors, or government agencies (for example, the FDA) for purposes such as quality control or safety.

What if you want to stop before your part in the study is complete?
You can withdraw from this study at any time, without penalty. The investigators also have the right to stop your participation at any time. This could be because the entire study has been stopped. If you withdraw from the study, you will receive the standard study incentive, as described below.

Will you receive anything for being in this study?
You will receive one item (a snack, beverage, or a household good) and cash for a total value of $40. The total value of the item you receive from the UNC Mini Mart will be deducted from the starting value of $40. For example, if your item is worth $3, you would receive $37 in cash, for a total value of $40. To participate in this study, you will always take one item home from the mini mart; therefore, you will always receive less than $40 in cash. We will also provide a voucher so that you do not need to pay for parking.

Will it cost you anything to be in this study?
It will not cost you anything to be in this study. You may have to pay for transportation to the study office. You will not be reimbursed for this cost, but you will receive a parking pass if needed.

Who is sponsoring this study?
This research is funded by Healthy Eating Research, a program of the Robert Wood Johnson Foundation.

What if you have questions about this study?
You have the right to ask, and have answered, any questions you may have about this research. If you have questions about the study (including payments), complaints, concerns, or if a research-related injury occurs, you should contact the researchers listed on the first page of this form.

What if you have questions about your rights as a research participant?
All research on human volunteers is reviewed by a committee that works to protect your rights and welfare. If you have questions or concerns about your rights as a research subject, or if you would like to obtain information or offer input, you may contact the Institutional Review Board at 919-966-3113 or by email to IRB_subjects@unc.edu.

Participant's Agreement:

I have read the information provided above. I have asked all the questions I have at this time. I give my verbal consent to participate in this research study.

Printed Name of Research Participant

____

Signature (RA)	Date
