# Supplementary material for: The impact of pictorial health warnings on purchases of sugary drinks for children: A randomized controlled trial
Source: PLoS Med. 2022 Feb 1;19(2):e1003885. doi: 10.1371/journal.pmed.1003885 (PMC8806063; doi:10.1371/journal.pmed.1003885)
Supplement: S1 Analytic Plan — (PDF) [file pmed.1003885.s006.pdf]

Impact of Warnings on Sugar-Sweetened Beverages

Hypotheses and Analytic Plan

NCT #04223687

Version Date: 1/24/2020

Hypotheses

The goal of this study is to examine whether pictorial warnings on sugar-sweetened beverages (SSBs) are more effective than neutral labels at reducing parents' purchases of SSBs for their children. Thus, our primary hypothesis is that participants who see a pictorial warning on SSBs will be less likely to purchase an SSB for their child compared to participants who see a neutral label (H1).

We also aim to examine the impact of pictorial warnings on precursors to behavior change and on other variables important to policymakers. Thus, we also hypothesize that, compared to exposure a neutral label, exposure to a pictorial warning on sugar-sweetened beverages will lead to:

- Lower calories purchased from SSBs
- Lower number of SSBs purchased
- Lower intentions to give SSBs to child
- Greater likelihood of noticing the trial label
- Higher thinking about the harms of drinking SSBs
- Greater negative affective reactions
- Higher anticipated social interactions
- Greater likelihood of feeling more in control of healthy eating decisions
- Higher perceived amount of added sugar in SSBs
- Higher perceived likelihood of child experiencing health problems due to SSBs
- Lower perceived healthfulness of SSBs for child
- Lower appeal of SSBs for child
- Lower perceived tastiness of SSBs for child
- Higher injunctive norms about limiting child's SSBs

Main analyses

We will use a two-sided critical alpha of 0.05 to conduct all statistical tests. All confidence intervals presented will be 95% and two-sided. Analyses of the primary and secondary outcomes will include all randomized participants according to the trial arm they were randomized to receive (i.e., intent-to-treat). We will use complete case analysis to handle any missing data.

To prepare the data, we will first examine all scales to ensure adequate internal consistency (i.e., Cronbach's alpha > 0.70), dropping items as needed to improve consistency. If we are unable to achieve adequate internal consistency by dropping items, we may exclude the unreliable scales from analyses (e.g., not analyze treatment effects on these outcomes).

We will descriptively report unadjusted means and percentages for the primary and secondary outcomes. For significance testing of our hypotheses, we will run chi-squared tests for dichotomous variables and independent samples t-tests for continuous variables.

We will also report results when controlling for any participant demographic characteristics found to be unbalanced across treatment arms in balance tests, if these results differ substantively from unadjusted results (i.e., changes in statistical significance or direction of effect). These analyses will use linear regression for continuous outcomes, logistic regression for dichotomous outcomes, two-part models for zero-inflated outcomes (e.g., SSB calories) and a count model for the number of SSBs purchased.

Exploratory analyses of the primary outcome

We will examine whether the following participant characteristics moderate the effect of SSB health warnings on likelihood of purchasing SSBs:

a) Age category of parent (in years);
b) Age category of child (2-5 vs. 6-12);
c) Gender of parent (man vs. woman);
d) Gender of child (boy vs. girl);
e) Sexual orientation (gay, lesbian, or bisexual vs. not);
f) Race of parent (white vs. non-white);
g) Ethnicity of parent (Hispanic/Latino vs. non-Hispanic/Latino)
h) Low educational attainment (some college or less vs. college or more),
i) Nutrition Facts Panel use (will dichotomize to create roughly equivalent groups based on frequencies);
j) Frequency of needing help reading medical information (will dichotomize to create roughly equivalent groups based on frequencies);
k) Household income ($50,000 or more vs. less than $50,000);
l) Child's frequency of consuming SSBs (above vs. at or below the sample median);
m) Language participant took the survey in (Spanish vs. English)

To test whether these characteristics moderate the effect of SSB health warnings on SSB purchase likelihood, we will fit a series of logistic regressions models (one for each potential moderator), with trial arm, the moderator, and their interaction as predictors. We will probe significant interactions by calculating the marginal effect of health warnings on the outcome at different levels of the moderating variable. Moderation analyses will use a Bonferroni-corrected *p*-value.

Sample size and power

The primary objective of this trial is to evaluate the effect of SSB health warnings on parents' likelihood of purchasing an SSB for their child. We used G*Power3 to determine sample size needs for addressing this objective. We based this off of the most similar study to date in terms of methods and stimuli (Grummon et al. 2019 Am J Prev Med), which found an effect size of d=.32 for the effect of warnings vs. control on likelihood of purchasing SSBs. Using these specifications and a two-sided alpha of 0.05, we determined a necessary sample of 314 to detect an effect of d=.32 or larger with 80% power. To account for potential missing data or incomplete study visits, we aim to enroll 326 participants (163 in each arm).

Interim analysis

No interim analyses are planned.
